# A heterozygous USB1 variant linked to immunodeficiency

**DOI:** 10.70962/jhi.20250110

**Published:** 2025-10-22

**Authors:** Alice Valagussa, Nidia Moreno-Corona, Chantal Lagresle-Peyrou, Sara Mercurio, Margot Tragin, Nicolas Goudin, Mélanie Parisot, Monica Beltrame, Despina Moshous, Sven Kracker

**Affiliations:** 1 Université Paris Cité, Laboratory of Human Lympho-hematopoiesis, Imagine Institute, INSERM UMR 1163, Paris, France; 2 Université Paris Cité, Laboratory of Lymphocyte Activation and Susceptibility to EBV infection, INSERM UMR 1163, Imagine Institute, Paris, France; 3 Paris Cité University, Imagine Institute, Paris, France; 4 Biotherapy Clinical Investigation Center, Groupe Hospitalier Universitaire Ouest, Assistance Publique – Hôpitaux de Paris, INSERM, Paris, France; 5 Dipartimento di Biotecnologie e Bioscienze, Università degli Studi di Milano-Bicocca, Milan, Italy; 6 Bioinformatics Facilities (Structure Fédérative de Recherche Necker), Imagine Institute, INSERM UMR 1163, Paris, France; 7 Structure Fédérative de Recherche Necker, INSERM-US24 CNRS-UAR3633, Necker Bioimage Analysis Platform, Paris, France; 8 Genomics Core Facility, Institut Imagine-Structure Fédérative de Recherche Necker, INSERM U1163 et INSERM US24/CNRS UAR3633, Paris Cité University, Paris, France; 9 Dipartimento di Bioscienze, Università degli Studi di Milano, Milan, Italy; 10 Department of Pediatric Immunology, Hematology and Rheumatology, Hôpital Universitaire Necker-Enfants Malades, Assistance Publique – Hôpitaux de Paris, Université Paris Cité, Paris, France; 11 Université Paris Cité, Laboratory of Genome Dynamics in Human diseases, Equipe Labellisée Ligue Contre le Cancer, Ligue 2023, Imagine Institute, INSERM UMR 1163, Paris, France; 12 Centre de Référence des Déficits Immunitaires Héréditaires, Necker-Enfants Malades University Hospital, Assistance Publique – Hôpitaux de Paris, Paris, France

## Abstract

Poikiloderma with neutropenia is a genetic disorder characterized by skin abnormalities, nail dystrophy, bone anomalies, and neutropenia. *USB1* encodes a phosphodiesterase essential for processing spliceosomal U6 RNA and some microRNAs, regulating their stability. This study describes a heterozygous *de novo* USB1 variant (p.P44L) identified in a patient with recurrent infections, hypogammaglobulinemia, and low neutrophil counts. Unlike previously reported mutations, p.P44L affects a conserved proline in the N-terminal domain, predicted to be critical for protein interactions and stability. Functional assays revealed that while U6 RNA processing remained intact, the variant altered protein interactions and subcellular localization, reducing nuclear presence and accumulation within nuclear speckles. *In vitro*, the variant did not prevent neutrophil differentiation but reduced clonal capacity. In zebrafish, it led to reduced neutrophils and pigmentation. These findings expand the spectrum of genetic traits associated with *USB1* and suggest that a heterozygous variant affecting the N-terminal domain of USB1 impacts clinical phenotypes and that hypogammaglobulinemia may be associated with USB1 dysfunction.

## Teaser

This study identifies a novel heterozygous USB1 variant (p.P44L) in a patient with hypogammaglobulinemia and low neutrophil counts, showing altered protein localization and function. Functional assays and zebrafish models reveal its impact on immune cells and pigmentation, expanding USB1-related disease understanding.

## Introduction

Poikiloderma with neutropenia (PN, OMIM #604173, also known as Clericuzio PN through the first description of the disease in 1991 by Clericuzio) is a disease characterized by genodermatosis, poikiloderma, pachyonychia, hyperkeratosis, bone anomalies, and neutropenia, predisposing to myelodysplasia (1, 2). Different disease-causing homozygous or compound heterozygous loss-of-function (LOF) mutations affecting the U6 small nuclear RNA (snRNA) biogenesis phosphodiesterase 1 (*USB1*) gene (aliases Mpn1 and C16orf57) have been identified in unrelated patients (3, 4, 5). *USB1* encodes a conserved phosphodiesterase that processes the 3′ end of spliceosomal U6 RNA (6) as well as certain microRNAs (7), thereby regulating their stability. In our study, we characterize a *de novo* heterozygous *USB1* variant identified in a primary immunodeficient patient.

## Results

### Clinical case and genetic investigations

**Figure 1.**
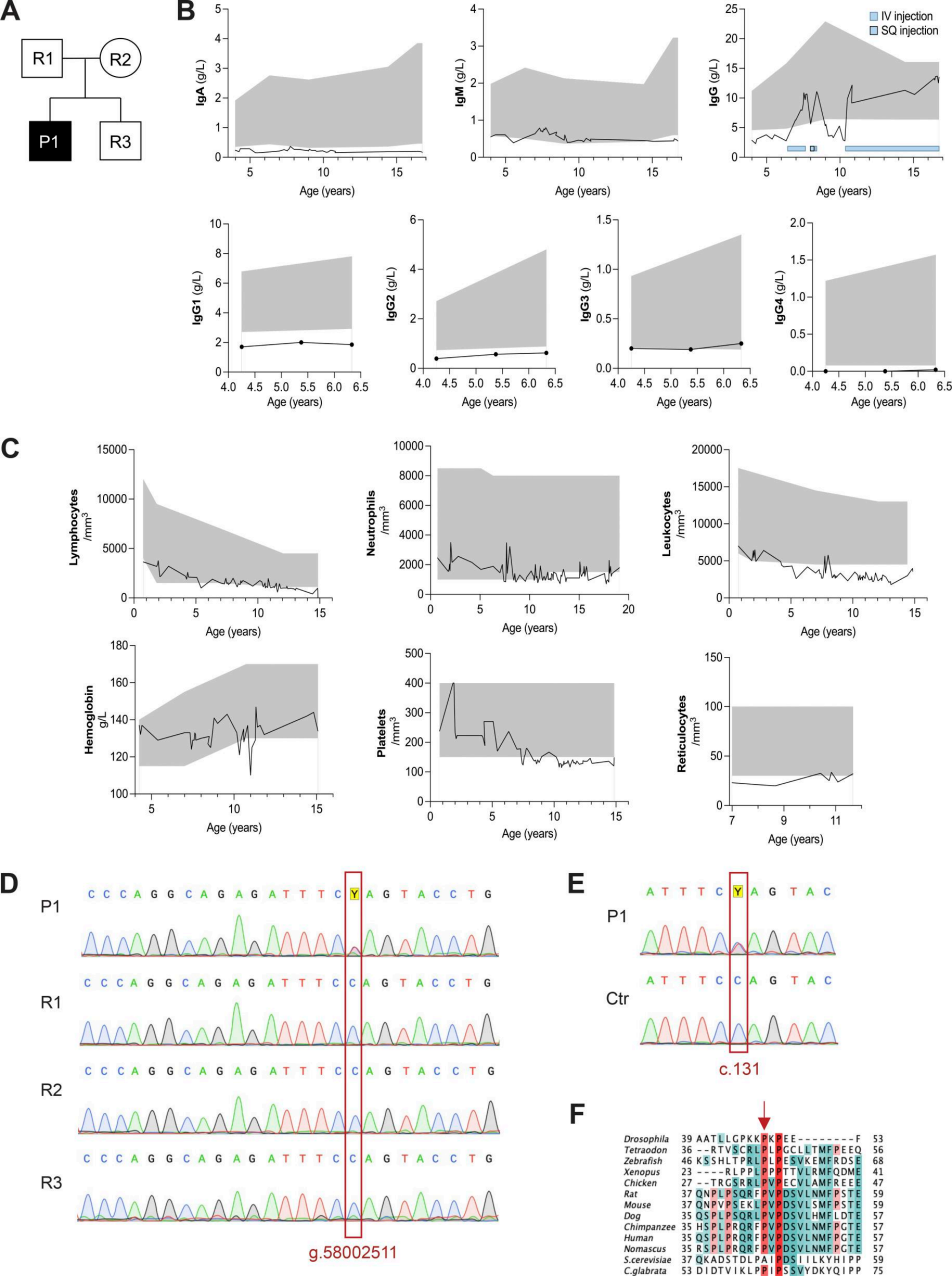
**Immunological manifestation of a patient carrying a *de novo USB1* heterozygous variant. (A)** The pedigree of the index family. **(B)** Serum IgA, IgM, IgG, IgG1, IgG2, IgG3, and IgG4 levels over time for the patient. **(C)** Neutrophils, leukocytes, lymphocytes, platelets, hemoglobin, and reticulocytes count over time for the patient. The grey area indicates the upper and lower reference boundaries across different ages. **(D and E)** Sanger sequencing analysis of gDNA from all family members (D) and *USB1* cDNA from LCLs derived from the patient (P1) and a healthy donor (Ctr) (E). **(F)** Protein alignment of human USB1 with orthologs. The red arrow indicates the location of the residue mutated in the patient (P44).

The patient, born to healthy, non-consanguineous parents, has suffered from gastroesophageal symptoms since birth and has received symptomatic treatment (Fig. 1 A). At 2 years of age, he developed septic arthritis of the right hip due to *Kingella kingae* infection. Around the same time, he started to present with recurrent ear, nose, and throat infections, resulting in a diagnosis of hypogammaglobulinemia (IgG = 2.78 g/l; IgA = 0.18 g/l; IgM = 0.53 g/l) at 4 years of age (Fig. 1 B). Serological testing revealed low antibody titers against diphtheria toxoid (0.5 IU/ml), tetanus toxoid (0.8 IU/ml), and poliovirus (1.4 IU/ml), whereas the response to *Streptococcus pneumoniae* was preserved (145 mg/ml). Ig replacement therapy was initiated at the age of 6 years, leading to a general clinical improvement. Over the years, he suffered from abdominal pain and diarrhea. Ileocolonoscopy at 6 years of age showed discrete inflammatory lesions in the duodenal mucosa without villous atrophy and an increase of intraepithelial lymphocytes. The patient presented with urticaria pigmentosa–type skin lesions, consistent with cutaneous mastocytosis diagnosed at 6 years of age. Recurrent anal pruritus and aphthous ulcers were also noted. Lymphocyte, neutrophil, leukocyte, and reticulocyte counts decreased with age and were below the normal range after the age of ∼12 years (Fig. 1 C). Hemoglobin and platelet levels were within the normal range. Immunophenotyping at central hospital facilities indicated normal distribution and ratio of CD4, CD8 T cell subsets, and B cell subsets at different ages. However, a low count of CD3, CD4, and CD8 T cell subsets was found (Table 1). The patient presented with scoliosis and has been wearing a corset since the age of 15 years. No neurocognitive defects have been reported. To identify possible genetic factors explaining the patient’s condition, whole-exome sequencing (WES) of DNA from total blood samples of the patient and both parents was performed. An autosomal recessive filter was applied to identify uniparental isodisomy, compound heterozygous, or homozygous variants. However, no likely disease-associated variant was identified. By analyzing the data with a *de novo* model, we identified the *USB1* (NM_024598) (alias Mpn1 and C16orf57) p.P44L, c. 131C>T variant. The variant, detected in 51% of *USB1* WES reads (Ref/Alt: 64/69), was exclusive to the patient and was neither recorded in our internal database (containing 24,284 exomes and 8,623 genomes, March 2025) or in several open-access databases for human genetic variations, such as the Exome Sequencing Project, the Exome Aggregation Database, and the Genome Aggregation Database. Sanger sequencing of the genomic DNA derived from peripheral blood confirmed its presence in a heterozygous state (Fig. 1 D). The USB1^P44L^ variant had a Combined Annotation Dependent Depletion score of 32 and damaging PolyPhen and Sorting Intolerant from Tolerant prediction scores of 0.9 and 0, respectively (8). Since the patient presented with a decreased number of neutrophils and neutrophil development is impaired in autosomal recessive USB1 deficiency, we further investigated the USB1^P44L^ variant. Analysis of the complete coding sequence of USB1 mRNA in a patient-derived Epstein-Barr virus–immortalized lymphoblastoid cell line (LCL) indicated the expression of both the variant and the wild-type USB1 allele, with no additional genetic aberrations detected (Fig. 1 E).

**Table 1.** Immunophenotyping of the patient at different years of age

​	Patient		Ref.	Patient	Ref.	Patient		Ref.
Age at evaluation (years)	4	6	3–6	10	7–12	14	17	13–18
T lymphocytes	​	​	​	​	​	​	​	​
CD3^+^ (/μl)	**1,304**	**1,043**	1,400–3,700	1,260	1,200–2,600	**916**	**672**	1,000–2,200
CD3^+^CD4^+^ (/μl)	775	**575**	700–2,200	780	650–1,500	**476**	**365**	530–1,300
Naive CD4^+^ CD45RA^+^ %	NA	**24.5**	53–86	NA	46–77	**39**	40	33–66
Memory CD4^+^ CD45RO^+^ %	NA	24.5	9–26	NA	13–30	29.5	35	18–38
T reg CD4^+^ CD25^+^CD127^LOW^ %	NA	4.6	4.2–12.5	NA	4.2–12.5	NA	NA	4.2–10.5
CD8^+^ (/μl)	**454**	**378**	490–1,300	490	370–1,100	**297**	**221**	330–920
Naive CD8^+^ CCR7^+^CD45RA^+^ %	NA	69.7	52–68	NA	40–75	51	**48**	52–68
Central memory CD8^+^ CCR7^+^CD45RA^−^ %	NA	**1**	12–30	NA	13–37	**4**	**5**	10–31
Effector memory CD8^+^CCR7^−^CD45RA^−^ %	NA	10.6	1.5–13	NA	1.5–15	**29**	**28**	2.3–15
Terminal effector CD8^+^CCR7^−^CD45RA^+^ %	NA	**39.6**	1.7–24	NA	2–21	16	19	5–31
Natural killer cells	​	​	​	​	​	​	​	​
CD3^−^CD56^+^ (/μl)	**114**	**91**	130–720	120	100–480	NA	NA	70–480
B lymphocytes	​	​	​	​	​	​	​	​
CD19^+^ (/μl)	397	**161**	273–860	350	219–509	226	163	193–628
Naive B cells CD27^−^IgD^+^ %	NA	75	59.7–88.4	NA	58.5–84.6	89	79	61.6–87.4
Memory B cells CD27^+^ %	NA	NA	8.1–33.3	NA	9–35	7	18	14.6–26.6
Unswitched memory CD27^+^IgD^+^ %	NA	12	3.1–18	NA	3–21.1	4.4	12	2.6–13.4
Switched memory CD27^+^IgD^−^ %	NA	10	2.9–17.4	NA	4.4–20.5	**3**	6	4–21.2
Transitional CD24^++^CD38^++^ %	NA	**12**	5.4–9.2	NA	4.5–9.2	**3**	7	3.9–7.8
Plasmablast CD24^−^CD38^++^ %	NA	**0**	0.8–2.7	NA	0.7–3.5	0.6	0.4	0.3–1.7
CD19^+^ CD21^−^CD38^−^ %	NA	2	1.8–5.2	NA	0.9–3.5	2	3	0.9–3.3

Summary of immunophenotyping results from the hospital laboratory. NA, not available. Values above or below reference (Ref.) ranges are marked in bold.

#### USB1^P44L^ retains U6 processing activity

**Figure S1.**
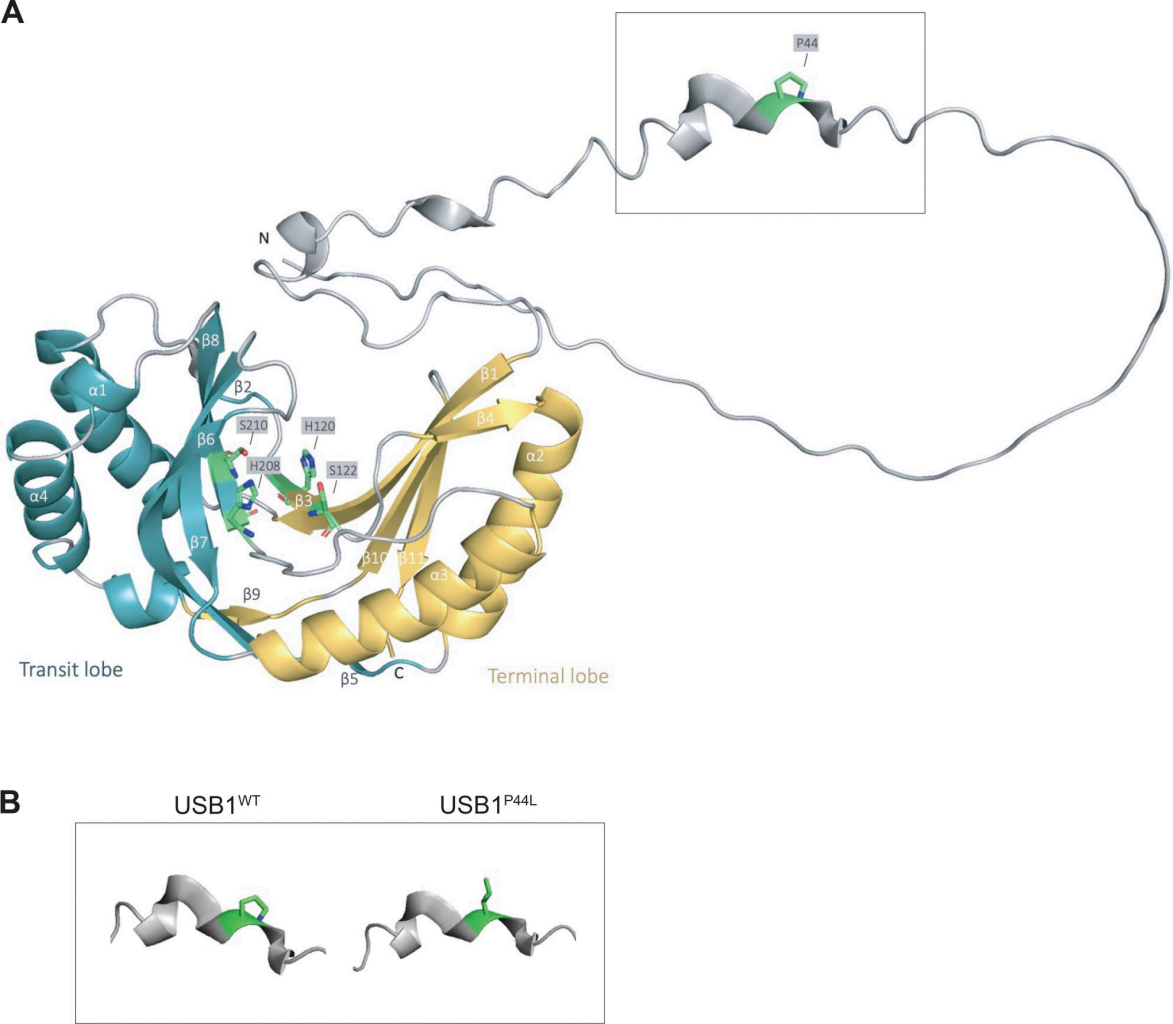
**Structural analysis of the P44L mutation in the USB1 protein. (A)** The *de novo* variant is situated within the only modeled ternary structure of the N-terminal domain, which is predicted to be predominantly disordered (AF-Q9BQ65-F1-v4, AlphaFold Protein Structure Database [10, 11, 12]). **(B)** Comparison of the local structural environment around residue 44 in the wild-type (USB1^WT^) and mutant (USB1^P44L^) proteins. The model was visualized using the PyMOL Molecular Graphics System (Version 3.1.0 Schrödinger, LLC).

**Figure 2.**
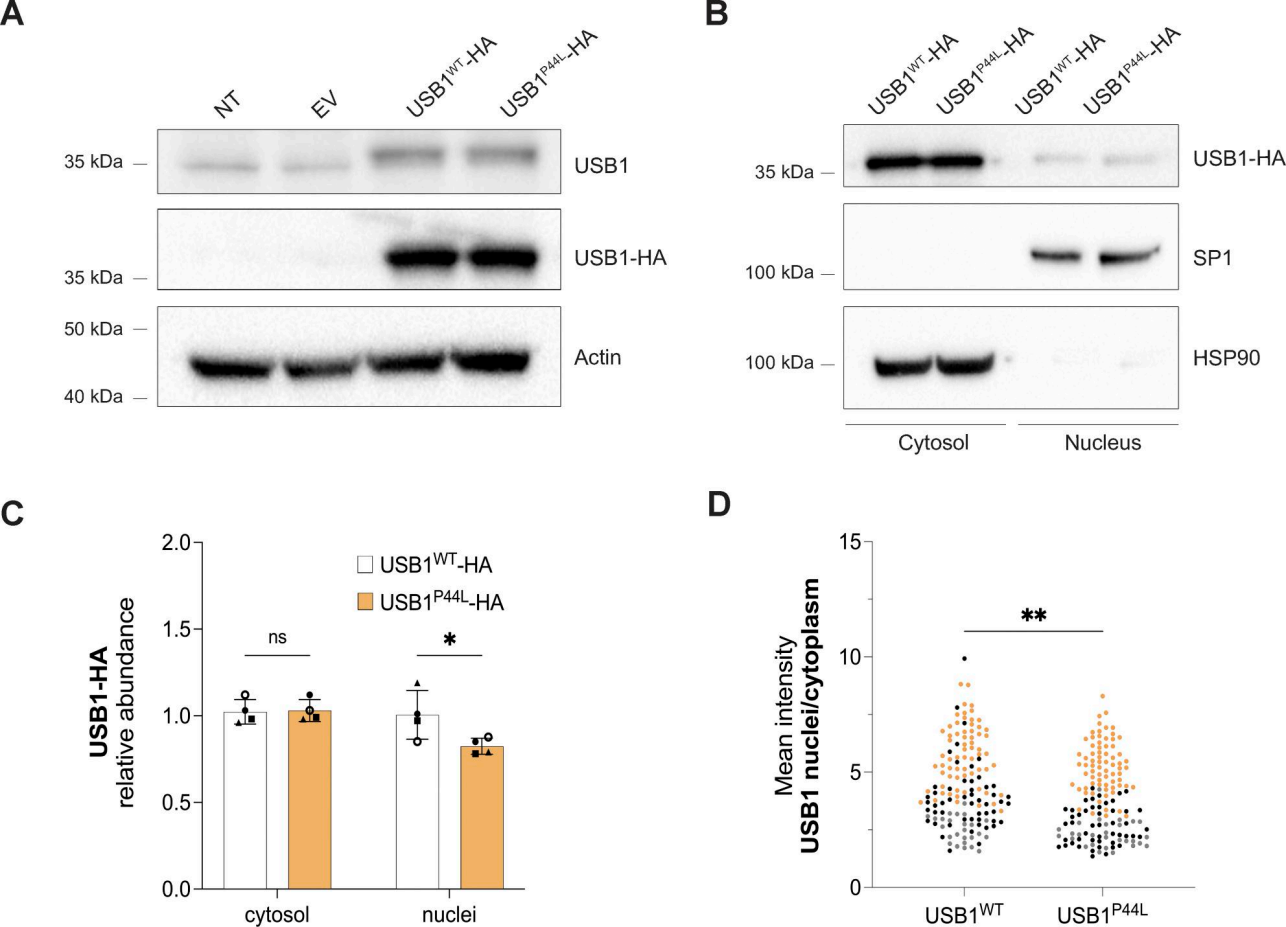
**USB1** ^**P44L**^ **has an altered subcellular localization. (A–C)** Western blot analysis of total cell (A) and cytosol vs. nucleus lysates (B and C) obtained from HEK293T cells ectopically expressing USB1-HA variants. Bars and error bars are averages of USB1-HA relative abundance normalized to HSP90 (cytosol) and SP1 (nucleus), and SD from four independent experiments. Two-way ANOVA statistical analysis was performed. “ns,” nonsignificant differences (P ≥ 0.05), *P < 0.05 (*n* = 4). **(D)** Semiautomatic quantification of immunofluorescence staining confirmed a decreased mean intensity nuclear vs. cytosolic ratio for HEK293T cells expressing USB1^P44L^ protein. Each independent experiment (*n* = 3) was represented with a different color. A minimum of 25 cells were analyzed for each independent experiment. An unpaired *t* test was performed. **P < 0.01. Source data are available for this figure: SourceData F2.

**Figure 3.**
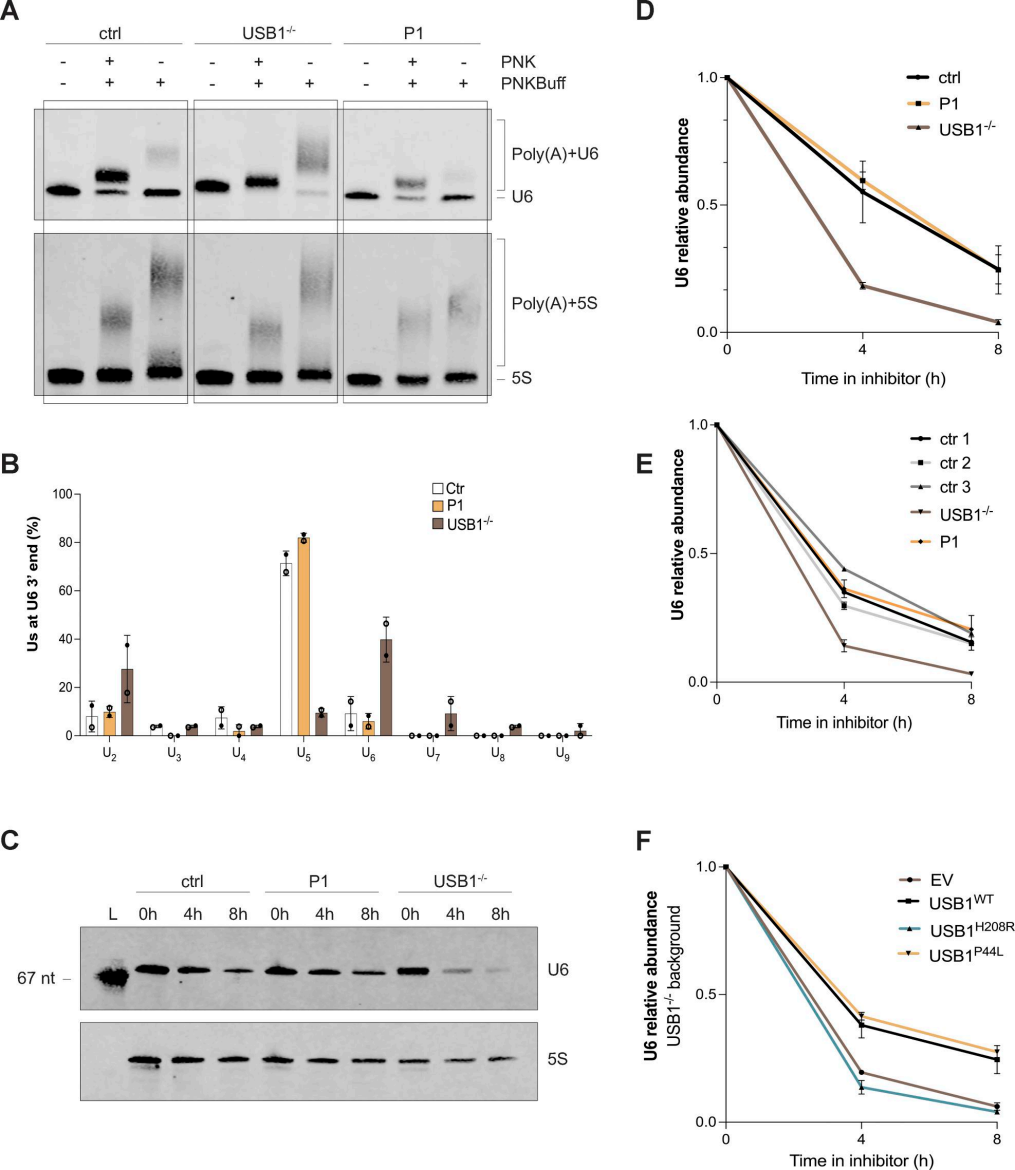
**The USB1 *de novo* variant is catalytically active and correctly processes U6 snRNA. (A)** Total RNA extracted from the indicated BEBV cell lines were treated with T4 PNK or with buffer only (PNKBuff) in mild acidic conditions. RNA was subsequently treated with poly(A) polymerase (PAP). Nontreated RNA was loaded as a control, (*n* = 2). **(B)** 3′ RACE analysis of U6 oligo(U) tails in the indicated cell lines. At least 24 clones per sample in each experiment (*n* = 2) were sequenced. Bars and error bars are averages of the number of U's within U6 oligo(U) tails and SEM from two independent experiments. **(C and D)** Indicated cell lines were treated with actinomycin D for 0, 4, and 8 h. RNA samples were processed by northern blotting for detection of U6 and 5S (*n* = 2). L: marker of known length (67 nucleotides). U6 signals were normalized through the corresponding 5S signals and successively expressed as fold decrease over U6 signal at time 0. Error bars are averages of SEM from two independent experiments. **(E and F)** U6 relative abundance quantification by qPCR analysis on patients and control cell lines (ctr1 *n* = 2, ctr2 *n* = 3, ctr3 *n* = 1, USB1^−/−^*n* = 3, P1 *n* = 3) (E), and USB1^−/−^ cells transduced with the indicated lentiviral constructs (*n* = 2) (F). U6 signals were normalized through the corresponding 5S signals and successively expressed as fold decrease over U6 signal at time 0. Error bars are averages of SEM from two independent experiments. EV, empty vector. Source data are available for this figure: SourceData F3.

The USB1^P44L^ variant affects an evolutionarily conserved proline within the N-terminal proline-rich domain of USB1 (Fig. 1 F and Fig. S1). Investigations in yeast using *USB1* truncation variants have shown that the N-terminal region of USB1 in both yeast and humans is essential for maintaining protein stability (9). However, the mechanism of how the N terminus of USB1 influences its stability remains unclear (9). To assess the USB1^P44L^ variant’s effect on protein stability, we ectopically expressed C-terminal HA-tagged USB1^WT^ and USB1^P44L^ variant in HEK293T cells. Western blot analysis of total cell lysates indicated a similar abundance of USB1^WT^ and USB1^P44L^ proteins (Fig. 2 A). Both were detected in the cytoplasm and nucleus (Fig. 2, B–D). However, the USB1^P44L^ protein was less abundant in the nucleus compared to USB1^WT^ (Fig. 2, C and D). In USB1 deficiency, the 3′ end processing of U6 snRNA is disrupted, leading to a decreased U6 half-life (6) (Fig. 3, A–F). In contrast, 3′ end processing and U6 snRNA stability were comparable in the patient-derived LCL and the control (Fig. 3, A–E). Lentiviral expression of the USB1^P44L^ variant in a USB1^−/−^ LCL increased U6 snRNA stability similar to lentiviral-expressed USB1^WT^ in contrast to an empty vector or a USB1^H208R^ LOF variant (Fig. 3 F). Together, these results suggested that the USB1^P44L^ variant retains U6 3′ end processing catalytic activity.

#### USB1^P44L^ affects the proteomic interactome

**Figure 4.**
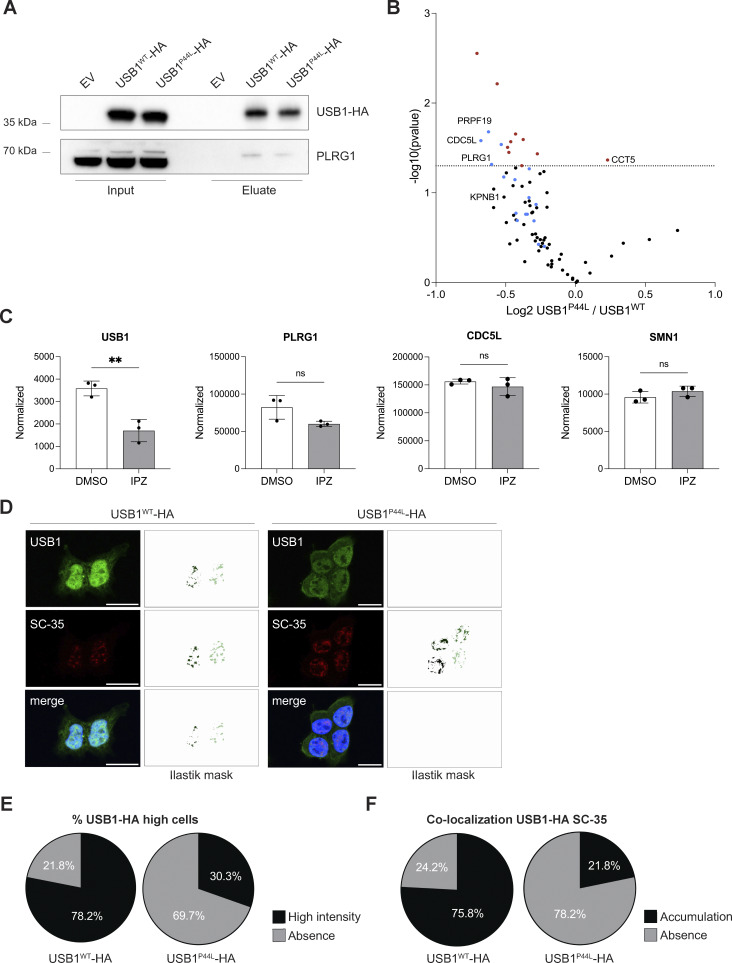
***USB1* *de novo* variant impacts USB1 protein interactome. (A)** HEK293T-USB1-HA lysates were immunoprecipitated using magnetic bead-bound anti-HA antibodies and analyzed by immunoblotting to validate the co-immunoprecipitation mass spectrometry results (*n* = 4). EV, empty vector. **(B)** Volcano plot of co-immunoprecipitation mass spectrometry experiments performed with HEK293T cells overexpressing USB1-HA variants (*n* = 4). Red dots denote statistically significantly enriched proteins. Known USB1 interactors are marked in blue. **(C)** Mass spectrometry analysis of nuclear fraction of DMSO- and IPZ-treated NB-4 cells (*n* = 3) (17). Proteomics data were obtained at PRIDE (PRoteomics IDEntification Database) under accession number PXD056172. An unpaired *t* test was performed for USB1 and its interactors (PLRG1, CDC5L, and SMN1). ns, nonsignificant differences (P ≥ 0.05), **P < 0.01. **(D)** Representative confocal microscopy images for HEK293T cells stably expressing USB1^WT^ or USB1^P44L^ variant (*n* = 3). SC-35 was included to visualize the nuclear speckles. Scale bar = 15 µm. For each image, pixel classification was performed using a machine learning Ilastik model, and the resulting mask is displayed. For the USB1-HA signal, we trained the model to create a mask of high-intensity signal voxels. **(E)** Percentage of cells presenting USB1-HA high-intensity signal in the nuclei (*n* = 3). **(F)** Signal overlap between USB1-HA and SC-35 was quantified using 3D pixel classification via an Ilastik machine learning model, focusing on high-intensity USB1-HA pixels. A threshold of 27 voxels was applied to define a cellular compartment. Semiautomated quantification was executed in Fiji (RRID:SCR_002285). IPZ, importazole. Source data are available for this figure: SourceData F4.

**Figure S2.**
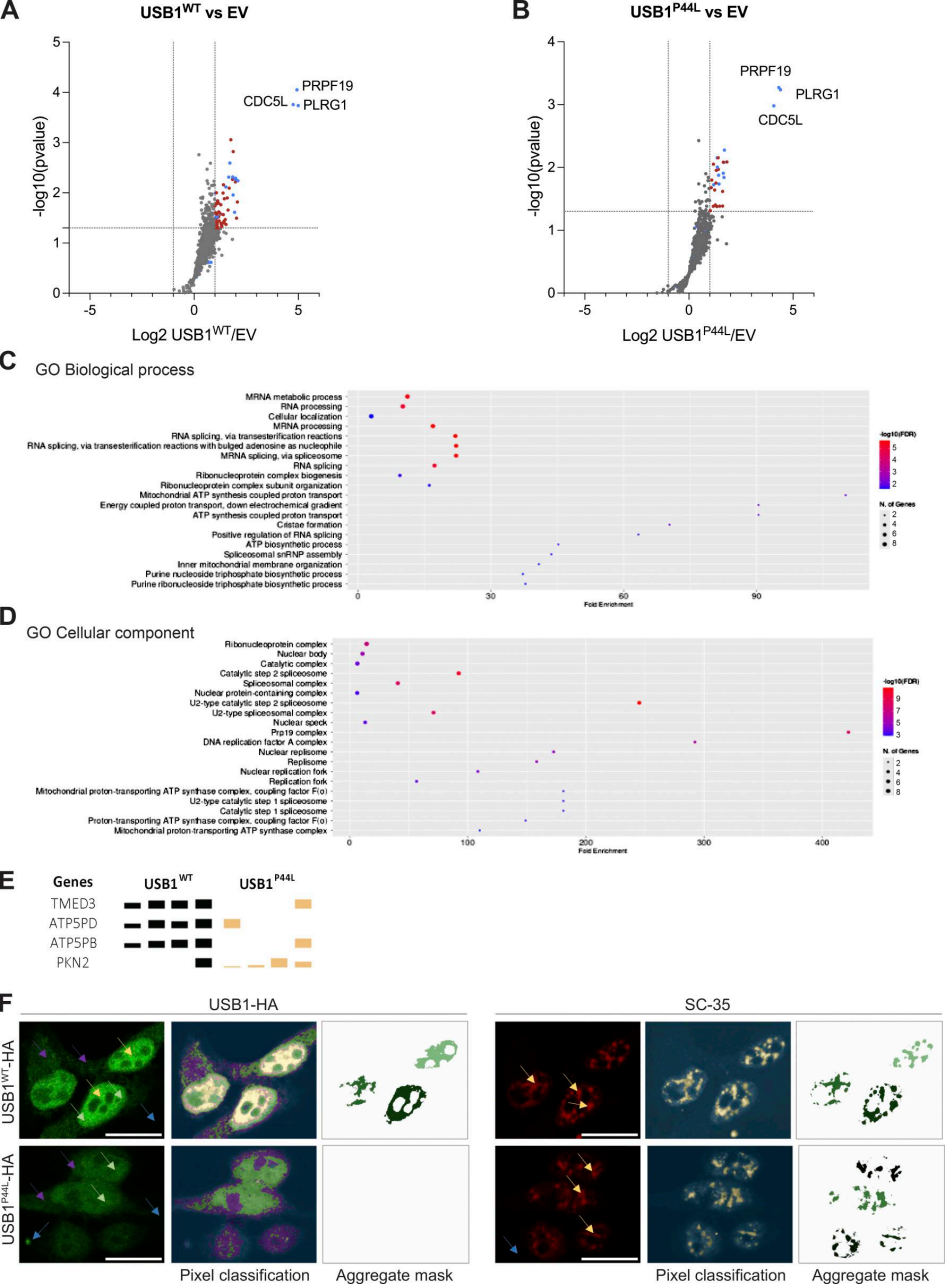
**USB1** ^**WT**^ **and USB1** ^**P44L**^ **interactome compared to the empty vector (EV), gene ontology (GO) enrichment analysis, and immunofluorescence pixel quantification. (A and B)** Volcano plot of USB1^WT^ (A) and USB1^P44L^ (B) interactome compared to the EV (*n* = 4). Red dots denote statistically significantly enriched proteins. Already known USB1 interactors are marked in blue. **(C and D)** GO Biological Process (C) and GO Cellular Component (D) were performed with ShinyGO v0.741 with P value cutoff (FDR) of 0.05 (18). **(E)** Reproducibly precipitated proteins not included in the volcano plot. Depicted are the four repetitions for each sample. The height of the bars indicates the detected amount. **(F)** Representative confocal microscopy images for HEK293T cells stably expressing USB1^WT^ or USB1^P44L^ variant (*n* = 3). Scale bar = 15 µm. To identify only USB1-HA high-intensity signal (left panel), four different labels were used to train the Ilastik machine learning model: high (yellow arrow), medium (green arrow), low (purple arrow), and background (blue arrow). Pixel classification for the SC-35 signal (right panel) was based on two labels: signal (yellow arrow) and background (blue arrow).

To investigate the impact of the USB1^P44L^ variant on protein–protein interactions, we performed immunoprecipitation experiments on HEK293T cells ectopically expressing HA-tagged USB1^WT^ and USB1^P44L^ proteins via magnetic bead-bound anti-HA antibodies (Fig. 4 A). Proteomic analysis identified a total of 87 proteins associated with USB1^WT^ and 40 proteins associated with USB1^P44L^ compared to the empty vector control (Fig. S2, A and B, and Table S1). Among these, 31 proteins were enriched in both conditions, including 12 previously reported USB1 interactors, such as PRPF19, CDC5L, and PLRG1 (13), validating our approach. Comparative analysis revealed 18 differently interacting proteins between USB1^WT^ and USB1^P44L^ interactomes (Fig. 4 B and Table S1). Gene ontology enrichment analysis indicated that PRPF19, CDC5L, PLRG1, RBM22, RBM27, DHX8, MTREX, RPL18, SNRPD3, and MYH10 are associated with RNA splicing/processing and ribonucleoprotein complex biogenesis, with additional associations to the spliceosome, PRP19 complex, and nuclear speckles (Fig. S2, C and D). The abundance of most proteins was reduced in the USB1^P44L^ interactome. Three proteins (TMED3, ATP5PD, and ATP5PB) were reproducibly precipitated only in the USB1^WT^ (Fig. S2 E). Notably, ATP5PD and ATP5PB are subunits of mitochondrial complex V involved in the ATP biosynthesis process (14), aligning with prior observation of dual USB1 localization to the nuclei and mitochondria in yeast, where USB1 overexpression may compensate respiratory deficiency (15). Conversely, TCPE (encoded by *CCT5*) and PKN2 were enriched in the USB1^P44L^ pulldown (Fig. 4 B and Fig. S2 E). Studies with *Cct5* knockout mice highlighted the importance of the CCT5-encoded protein for hematopoietic stem cells homeostasis and differentiation into myeloid and lymphocyte compartments (16). KPNB1, a protein that mediates the docking of the importin/substrate complex to the nuclear pore and thus promotes nuclear import, was enriched in the USB1^WT^ interactome compared to USB1^P44L^ (Fig. 4 B). To date, there are no studies directly addressing USB1 transport mechanisms. By analyzing public proteomic data from a study investigating KPNB1-mediated cargo proteins (17), we observed reduced nuclear levels of USB1 in cells treated with importazole, a KPNB1 inhibitor (Fig. 4 C). Taken together with our previous observations of reduced nuclear localization of the USB1^P44L^ variant (Fig. 2, C and D) and the evidence of the KPNB1–USB1 interaction (Fig. 4 B), these findings suggested that KPNB1 plays a role in mediating the intracellular transport of USB1 into the nucleus.

Further immunofluorescent microscopy 3D analysis indicated the presence of a high-intensity signal within the nucleus, mainly in USB1^WT^ expressing cells (Fig. 4, D and E; and Fig. S2 F). This suggests that the USB1^P44L^ variant has an altered subcellular distribution, especially within the nucleus. The gene ontology analysis (Fig. S2, C and D) highlighted “nuclear speckles,” also referred to as nuclear splicing factor compartments. These subcellular structures can be visualized with an antibody directed against the spliceosome assembly factor splicing component 35 (SC35) (19). Immunofluorescent staining of SC35 and ectopically expressed C-terminal HA-tagged USB1^WT^ in lentiviral-transduced HEK293T cells indicated the presence of USB1 in nuclear speckles (Fig. 4, D and F). Decreased accumulation of USB1^P44L^ within nuclear speckles, largely associated to less high USB1 intensity signal, was observed. Together, our immunofluorescent and proteomic analysis indicated an impact of the USB1^P44L^ variant on the USB1 protein interactome.

#### *In vitro* and *in vivo* analysis of USB1^P44L^ function

**Figure S3.**
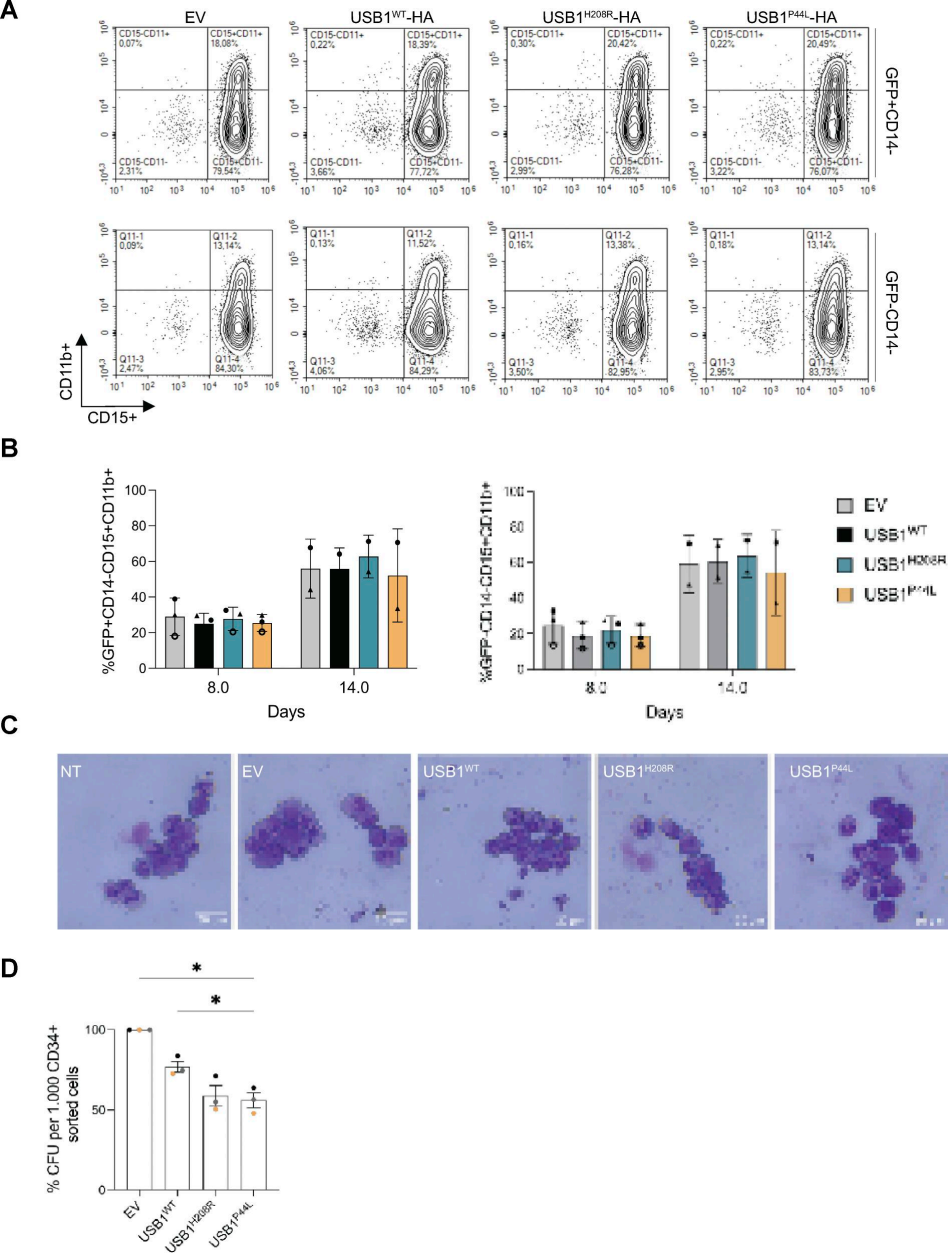
**USB1** ^**P44L**^ **expression does not block *in vitro* neutrophil differentiation of CD34** ^**+**^ **human cells. (A)** *In vitro* liquid culture differentiation (SCF, G-CSF) of CD34^+^ representative images at day 8. CD15^+^/CD11b^+^ plots are gated on GFP^+^ (up) and GFP^−^ (down) CD14^−^ cells for the indicated USB1 variants. **(B)** Percentage of CD14^−^CD15^+^CD11b^+^ cells either GFP^+^ (left) or GFP^−^ (right). Bars and error bars are the averages of the percentage of the indicated populations and SD from at least two independent experiments (*n* ≥ 2). **(C)** Representative MGG staining images of sorted GFP^+^ cells showing the characteristic morphology of neutrophils (day 14, *n* = 3). Scale bar = 20 µm. **(D)** CFU potential of myeloid differentiation in GFP^+^ sorted cells at 2.5 days after transduction (*n* = 3). Ordinary one-way ANOVA statistical analysis was performed. *P < 0.05. Nonsignificant differences were not annotated. CFU, colony-forming unit; EV, empty vector.

**Figure 5.**
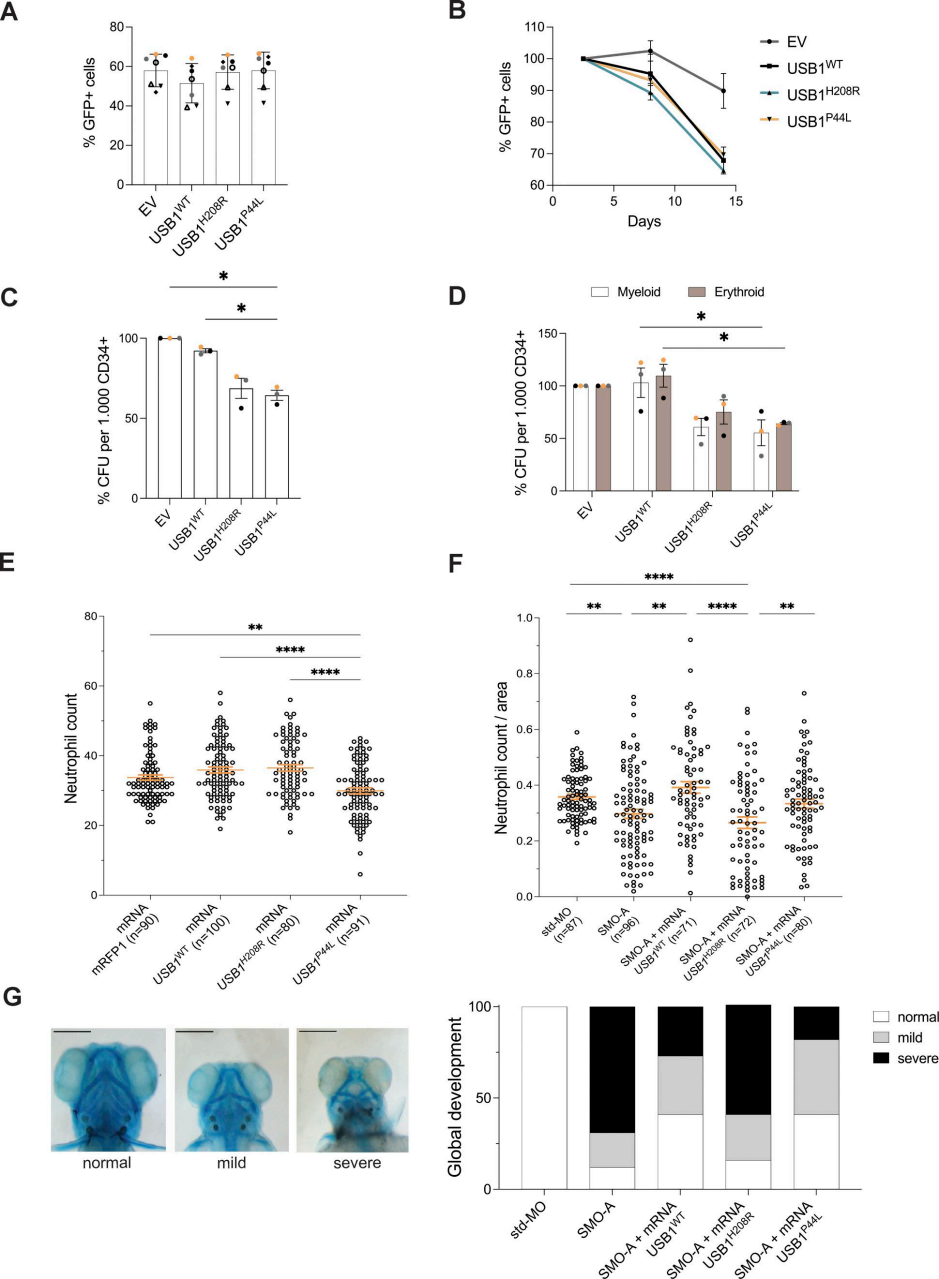
**The USB1** ^**P44L**^ **variant impacts myeloid differentiation *in vitro* and *in vivo*. (A)** Percentage of GFP^+^ cells in human CD34^+^ cells at day 2.5 following the transduction step (*n* = 7). Bars and error bars are the averages of the percentage of alive GFP^+^ cells and SD from seven independent experiments. **(B)** Percentage of GFP^+^ cells at days 8 and 14 normalized to the GFP^+^ population at day 2.5. Error bars are SEM from a minimum of two independent experiments (*n* = 2). **(C)** Colony-forming unit (CFU) potential of myeloid differentiation (*n* = 3). **(D)** CFU potential of myeloid (white bars) and erythroid (brown bars) differentiation (*n* = 3). (D) were evaluated after an 8-day culture. Bars and error bars are the averages of the percentage of the indicated populations and SEM from three independent experiments. Ordinary one-way ANOVA statistical analysis was performed. *P < 0.05. Statistically nonsignificant differences were not annotated. **(E)** Neutrophil count at 2 dpf in zebrafish overexpressing indicated *USB1* variant RNAs (*n* = 4 biological replicates, in orange mean ± SEM). Ordinary one-way ANOVA statistical analysis was performed. **P < 0.01 and ****P < 0.0001. Statistically nonsignificant differences were not annotated. **(F)** SMO-A morphants injected with indicated USB1 variants (*n* = 4 biological replicates, in orange mean ± SEM). Ordinary one-way ANOVA statistical analysis was performed on log_2_-transformed data. *P < 0.05, **P < 0.01, ***P < 0.001, and ****P < 0.0001. Statistically nonsignificant differences were not annotated. **(G)** Classification of Alcian blue staining highlights morphological alterations at 5 dpf (*n* = 3). From left to right: ventral view of a control embryo (left) and representative pictures of the mild (middle) and severe (right). Scale bar = 250 μm.

To evaluate the impact of the USB1^P44L^ on neutrophil differentiation *in vitro*, human CD34^+^ hematopoietic stem and progenitor cells (HSPCs) were transduced with lentiviral vectors coexpressing the different USB1 variants and the GFP reporter gene to follow the transduced cells. Cells were subsequently differentiated in liquid cultures into the neutrophil lineage. Once gated on CD14^−^ (GFP^+^ or GFP^−^ alive cells), the frequencies of CD15^+^/CD11b^+^ populations were not affected by the expression of the different USB1 variants, indicating that none of the USB1 variants blocked neutrophil differentiation either at day 8 or day 14 (Fig. S3, A and B). Additionally, May–Grünwald Giemsa (MGG) staining revealed no detectable morphological differences among experimental conditions (Fig. S3 C), further supporting that neutrophil maturation occurs across all groups. At day 2.5 after transduction, we found a similar percentage of GFP^+^ cells in infected cells (Fig. 5 A). However, over time (after 8 and 14 days of culture), cells ectopically expressing different USB1 variants, including the wild type, showed a decreased percentage of GFP^+^ cells (Fig. 5 B). This suggests that high levels of USB1 protein disadvantage the cells. To further assess the clonal capacity of cells harboring the various USB1 variants throughout the myeloid and erythroid lineages, human colony-forming unit assays with and without erythropoietin were performed. We observed reduced myeloid and erythroid colony formation in cells overexpressing USB1^H208R^ or USB1^P44L^ variants compared to USB1^WT^ (Fig. 5, C and D; and Fig. S3 D). Together, this indicated that while the USB1^P44L^ variant does not block neutrophil differentiation as such, it impacts the clonal capacity in the myeloid lineage.

**Figure S4.**
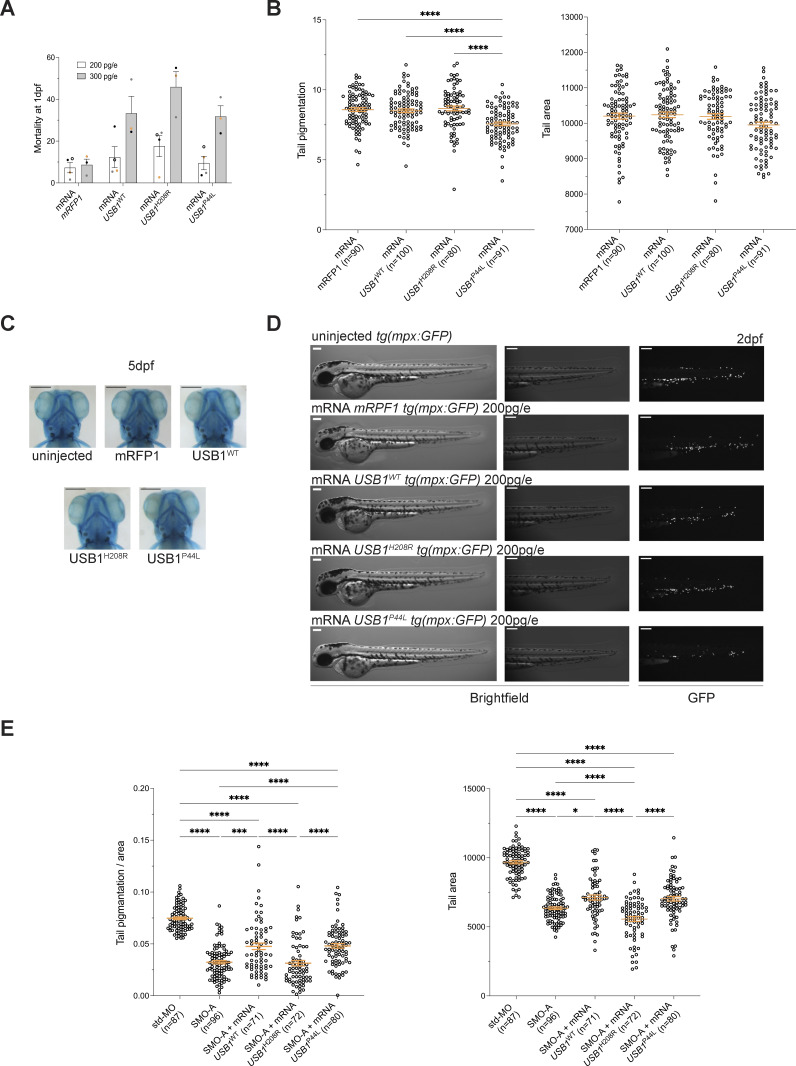
**Ectopic expression of USB1** ^**P44L**^ **in zebrafish. (A)** Injections of a higher dose of *USB1* mRNA (300 pg/e) were associated with increased mortality of the embryo at 1 dpf (*n* ≥ 3). Of note, no difference in the mortality between the two doses was registered when injecting the control *mRFP1* mRNA. Bars and error bars are averages of the percentage of mortality at 1 dpf and SEM from at least three independent experiments. **(B)** Tail pigmentation and area of the tail at 2 dpf of zebrafish overexpressing different USB1 variants (*n* = 4 biological replicates, in orange mean ± SEM). Ordinary one-way ANOVA statistical analysis was performed. ****P < 0.0001. Nonsignificant differences were not annotated. **(C)** Alcian blue staining at 5 dpf did not reveal any skeletal defects induced by overexpression of the different USB1 variants. Scale bar = 250 μm. **(D)** Lateral views live-microscopy 5× magnification pictures of *tg(mpx:GFP)* uninjected and injected embryos with 200 pg/e of the respective construct. Obvious morphological alterations are not reported in uninjected and injected embryos. USB1^P44L^-overexpressing embryos presented a decrease in neutrophil count in the tail. Scale bar = 250 μm. **(E)** Pigmentation normalized to the tail area and tail area of morphants expressing the different *USB1* mRNA variants (*n* = 4 biological replicates, in orange mean ± SEM). Ordinary one-way ANOVA statistical analysis was performed on log_2_-transformed data. *P < 0.05, ***P < 0.001, and ****P < 0.0001. Statistically nonsignificant differences were not annotated.

Next, we studied the functional impact of the USB1^P44L^ variant in zebrafish. A zebrafish model of PN, utilizing a morpholino-mediated *usb1* knockdown, has been described to recapitulate human syndrome hallmarks, including neutropenia (20). Using the *Tg(mpx:GFP)* zebrafish line, we injected human mRNA (200 pg/embryo) encoding USB1 proteins into embryos. Notably, injections of a higher dose of *USB1* mRNA variants (300 pg/embryo) increased mortality at 1 day postfertilization (dpf) (Fig. S4 A). At 2 dpf, embryos injected with the mRNA (200 pg/embryo) encoding the USB1^P44L^ variant showed reduced neutrophil count and pigmentation compared to those injected with mRNA encoding USB1^WT^, USB1^H208R^ LOF variant, and *mRFP1* mRNA (Fig. 5 E and Fig S4 B). No differences in tail area, morphology (2 dpf), or development (5 dpf) were observed across all groups (Fig. S4, B–D). Since the USB1^P44L^ variant retains U6 3′ end processing catalytic activity, we next investigated whether USB1^P44L^ could rescue the morpholino-mediated *usb1* knockdown phenotype. As reported, splice-blocking morpholino (SMO)-A morphants showed reduced neutrophil count and pigmentation at 2 dpf compared to controls (Fig. 5 F and Fig. S4 E). At 5 dpf, 80% of SMO-A morphants had defective pharyngeal arch architecture with varying degrees of severity (Fig. 5 G). Co-injection of human *USB1*^*WT*^ or *USB1*^*P44L*^ mRNA with SMO-A rescued the phenotype, increasing neutrophil count and pigmentation to near-control levels and improving overall development (Fig. 5 F and Fig. S4 E). However, co-injection with the catalytically dead USB1^H208R^ variant did not rescue these parameters. Together, these results suggested that while USB1^P44L^ retains functional activity, it can be detrimental regarding neutrophil count and pigmentation.

### Discussion

We have identified a specific heterozygous variant of *USB1*, leading to the expression of a USB1^P44L^ protein, which is likely to be associated with an inborn error of immunity in a patient who presented early in life with hypogammaglobulinemia, and in whom counts of lymphocytes, neutrophils, leukocytes, and reticulocytes decreased with age. The *de novo* USB1^P44L^ variant, which has never been reported in publicly available databases, affects an evolutionarily conserved proline residue within the proline-rich N-terminal domain of the USB1 protein. Functional evidence supporting the disease association of the USB1^P44L^ variant includes the following observations: (1) ectopic expression of the USB1^P44L^ variant in CD34^+^ HSPCs reduced myeloid and erythroid colony formation compared to USB1^WT^, and (2) injection of mRNA encoding the USB1^P44L^ variant in zebrafish reduced neutrophil count and pigmentation compared to controls.

Homozygous and compound heterozygous *USB1* LOF variants cause PN. Therefore, the *de novo* USB1^P44L^ variant is unlikely to be a LOF variant due to its disease association as a heterozygous variant. Indeed, the USB1^P44L^ variant retains its catalytic activity, as demonstrated by its ability to process U6 RNA similarly to the wild type and by the partial phenotypic rescue observed in the PN zebrafish model. Although neutropenia is one of the major manifestations in PN, a recent study enlarged the spectrum of affected cellular subsets by revealing that defective monocyte plasticity may contribute to disease manifestations in PN (21).

USB1^P44L^ may exert its pathogenic effect through altered interaction dynamics. Our proteomic data revealed a significant reduction in the number of interactors for the USB1^P44L^ variant compared to USB1^WT^. This supports the hypothesis that the highly conserved proline residue USB1^P44L^, mutated in the patient, plays a critical role in protein–protein interactions and complex formation. Our study indicated that KPNB1 was enriched in the USB1^WT^ interactome compared to USB1^P44L^, and nuclear USB1 levels decreased with KPNB1 inhibition (17). Together, these observations supported a role for KPNB1 in USB1 nuclear import and could explain the reduced nuclear localization of USB1^P44L^. Gene ontology enrichment analysis of proteins with decreased interaction with USB1^P44L^ highlighted pathways related to RNA splicing, RNA processing, ribonucleoprotein complex biogenesis, and nuclear speckles. Immunofluorescence staining reported that USB1 co-localizes with SC-35–marked nuclear speckles, while USB1^P44L^ displayed reduced accumulation, indicating an altered subcellular distribution.

A recent study proposed a novel and U6-independent role for USB1 in PN, suggesting that the USB1 function is important for miRNA stability (7). This regulation is thought to occur either through the steric inhibition of exonucleases or by catalyzing the formation of a 2′–3′ cyclic phosphate at the 3′ end of miRNAs. Both mechanisms could protect miRNAs from degradation and thereby modulate their abundance and functional activity within hematopoietic cells. Although our study did not investigate the impact of USB1^P44L^ on miRNA stability, it is noteworthy that many of the USB1 interactors are miRNA-binding proteins (22). The reduced interaction of the USB1^P44L^ variant with its interactors might result in the dysregulation of miRNA levels. Whether, how, and on which miRNAs USB1^P44L^ (and USB1^WT^) might act are interesting questions that should be addressed by future studies.

Our data, which involved the expression of various USB1 variants in human CD34^+^ HSPCs as well as zebrafish embryos, indicated that USB1 protein levels within cells have a limited dynamic range that is sufficient to support physiological USB1 function. Patients with partial duplication of chromosome 16q have been reported (23). Notably, the *USB1* gene is localized within the cytogenetic band 16q21. Based on our observations, one may speculate that increased USB1 protein levels due to chromosomal duplication involving 16q21 might contribute to clinical presentations such as recurrent episodes of respiratory tract infections in these patients.

Our patient presented early in life with hypogammaglobulinemia and intermittent neutropenia. Interestingly, a recent study also documented hypogammaglobulinemia alongside neutropenia in a Clericuzio-type PN patient (24). These cases highlight the importance of serum immunoglobulin monitoring in PN patients, as early detection of immunoglobulin deficiencies could enhance disease management and improve quality of life. Cutaneous mastocytosis, observed in our patient, has previously been reported in a PN patient (25), suggesting that this dermatological manifestation may be part of the broader phenotypic spectrum of PN.

The pathological mechanism of the USB1^P44L^ variant appears to involve altered cellular localization and protein interactions in contrast to the autosomal recessive form of USB1 deficiency (PN), in which U6/miRNA end processing is disease causing. However, we cannot exclude the contribution of additional genetic modifiers or environmental factors that may shape the clinical presentation, particularly the gastrointestinal symptoms and hypogammaglobulinemia observed in the patient.

In conclusion, our study indicates that disturbed USB1 function can arise independently of its catalytic activity due to a heterozygous variant affecting the N-terminal proline-rich domain of USB1.

### Materials and methods

#### Blood sample collection from patients and healthy donors

Peripheral blood samples were collected from the patient after they provided written, informed consent. Genetic studies and data collection procedures were approved by the local institutional review board (Comité de Protection des Personnes Ile de France II, Paris, France; reference: 2015-01-05; 2015-01-05 MS2) and the French Advisory Committee on Data Processing in Medical Research (Comité Consultatif sur le Traitement de l’Information en matière de Recherche dans le domaine de la Santé, Paris, France; reference: 15.297bis).

Genomic DNA was extracted from PBMCs. Exome libraries were performed with the Twist Bioscience kits (Twist Human RefSeq Exome Kit, 36 Mb) and with the protocol version Twist-NGS Exome-96-12-DOC-001016-Rev1.0-May2018. Briefly, genomic DNA (500 ng) was sheared with an Ultrasonicator (Covaris). A total amount of 50 ng of the fragmented and purified double-strand DNA was used to prepare Twist Exome libraries as recommended by the manufacturer, but with no initial enzymatic shearing and using adaptators with Unique Dual Identifier (IDT). Barcoded exome libraries were pooled and sequenced with the NovaSeq6000 system (Illumina), generating paired-end reads (100 bases + 100 bases). After demultiplexing, sequences were aligned to the reference human genome hg19 using the Burrows–Wheeler Aligner. The mean depth of coverage obtained was >111X with >97.9% of the targeted exonic bases covered by at least 15 independent reads and >97.3% covered by at least 30 independent reads. Downstream processing was carried out with the Genome Analysis Toolkit, SAMtools (RRID:SCR_002105), and Picard (RRID:SCR_006525), following documented best practices (http://www.broadinstitute.org/gatk/guide/topic?name=best-practices).

#### Cell line culture

The *USB1*^*−/−*^ BEBVs were provided by Elisa A. Colombo from Università degli Studi di Milano-Bicocca, Milan, Italy. The other BEBV cell lines were obtained from the Centre de Ressources Biologiques (Necker Campus). BEBVs were cultured in RPMI-1640 medium (#21875-034; Gibco) supplemented with 15% fetal bovine serum (FBS, #10270-106; Gibco) and 10 µg/ml Gentamicin (#15710-049; Gibco). 5 mg/ml Actinomycin D (#BML-GR300; Enzo) was added when indicated. HEK293T cells (RRID:CVCL_0063) were cultured in DMEM with GlutaMAX (#31966-047; Thermo Fisher Scientific), 10% FBS (#10270-106; Gibco), and 10 µg/ml Gentamicin (#15710-049; Gibco). All cell lines were tested negative for mycoplasma contamination.

#### gDNA and mRNA sequencing

gDNA was isolated from the peripheral blood of patient and parents using the FlexiGene DNA Kit (51206; Qiagen). A first PCR was performed using specific primers (USB1_gDNA_F 5′-ACC​CCA​ATG​AGA​CAA​TAC​TGG​A-3′ and USB1_gDNA_R 5′-GGT​GCC​CGG​GAA​CAT​GTT-3′) and GoTaq DNA Polymerase (#M7845; Promega). The correct size of the amplicon was confirmed by a resolution of products in a 1% agarose gel. Sequencing was performed using USB1_gDNA_F primer.

To sequence the mutation site, PCR amplification was performed with Go Taq G2 DNA (M784B; Promega), USB1_cDNA primers (USB1_F cDNA 5′-CTG​CTC​TGG​TGG​TCT​TGG​AT-3′ and USB1_R cDNA 5′-CCC​GTG​TTT​TGT​GCT​GTC​AT-3′), and a melting temperature (Tm) of 70°C. HPRT1 (HPRT1_F 5′-CCG​GCT​TCC​TCC​TCC​TGA-3′ and HPRT1_R 5′-TCT​CGA​GCA​AGA​CGT​TCA​GT-3′) amplification was used as an internal control (Tm 60°C). The forward primer was used for the sequencing reaction. All PCR products were used in the subsequent sequencing BigDye reaction (Terminator 3.1 Sequencing Kit Applied Biosystems) with the indicated primers. The sequence reaction was read using the Applied Biosystems 3500 Series Genetic Analyzer (Rapid_Seq_Assay_XL_POP7). Results were analyzed by SnapGene software (http://www.snapgene.com, RRID:SCR_015052).

#### Plasmids and lentiviral vectors

pCS2+ plasmid containing full-length human wild-type *USB1* was a kind gift from E. Colombo (Università degli Studi di Milano-Bicocca, Milan, Italy). Following the manufacturer’s instructions, the GeneArt Site-Directed Mutagenesis System (#A13282; Thermo Fisher Scientific) was utilized along with specific primers (USB1_131_CT_F 5′-CCA​GGC​AGA​GAT​TTC​TAG​TAC​CTG​ACA​GTG​T-3′, USB1_131_CT_R 5′-ACA​CTG​TCA​GGT​ACT​AGA​AAT​CTC​TGC​CTG​G-3′, USB1 623_AG_F 5′-AGG​ATC​CTT​CTT​TCC​GCC​TCA​GCC​TGG​CCT​G-3′ and USB1 623_AG_R 5′-CAG​GCC​AGG​CTG​AGG​CGG​AAA​GAA​GGA​TCC​T-3′) to introduce the mutations of interest. Those inserts (with the addition of an HA tag at the C-terminal when indicated) were subcloned into the lentiviral pWPI backbone (RRID:Addgene_12254) by GenScript. The Structure Fédérative de Recherche (SFR) BioSciences Gerland-Lyon Sud (Lyon, France) vector facility produced the lentiviral supernatant.

#### Transduction

BEBVs were lentivirally transduced with the different constructs (pWPI-EV RRID:Addgene_12254, USB1^WT^, USB1^H208R^, and USB1^P44L^) with 0.25 mg/ml LentiBOOST (SIRION BIOTECH) without antibiotics (multiplicity of infection [MOI] 30), for 6 h at 37°C. At day 12 after transduction, cells were resuspended in FACS buffer (PBS, 2% FBS, and 1 mM EDTA) with 7-AAD and sorted using a 100-µm nozzle (BD FACS Aria II SORP; SFR Necker). HEK239T cells (RRID:CVCL_0063) were lentivirally transduced with the different constructs (pWPI-EV, USB1^WT^-HA, and USB1^P44L^-HA) with 0.25 mg/ml LentiBOOST (SIRION BIOTECH) without antibiotics (MOI 20) for 6 h at 37°C. 0.2 M/condition of CD34^+^ cells were plated in 100 μl of pre-activation media. The day after, cells were transduced overnight with a MOI of 100 and 0.25 mg/ml of LentiBOOST (SIRION BIOTECH).

#### Cell lysis and western blot

Previously transduced HEK293T cells (RRID:CVCL_0063) were collected directly from culture flasks, washed once with PBS, and lysed in Cell Lysis Buffer (#9803; Cell Signaling). Cytosolic versus nuclear protein extraction was performed according to the manufacturer’s instructions (NE-PER Nuclear and Cytoplasmic Extraction Reagents, #78833; Thermo Fisher Scientific). After adding the nuclear extraction reagent (NER) buffer, samples were sonicated for 10 min at 4°C (Bioruptor Pico). Protease/Phosphatase Inhibitor Cocktail (1X) (#5872S; Ozyme) was added to the lysis buffers immediately before cell lysis. Proteins were quantified (Pierce Detergent Compatible Bradford Assay Reagent, #1863028; Thermo Fisher Scientific) and boiled with Bold LDS Sample Buffer 4× (B0008; Thermo Fisher Scientific) and β-mercaptoethanol (M3146; Sigma-Aldrich). Protein extracts (20 μg) were resolved using SDS-PAGE on a NuPAGE 12% Bis-Tris gel (#NP0342BOX; Invitrogen). A Spectra Multicolor Broad Range Protein Ladder (#26634; Thermo Fisher Scientific) was included for molecular weight reference. Proteins were transferred onto a low-fluorescence polyvinylidene difluoride membrane using the iBlot 3 Western Blot Transfer System (Thermo Fisher Scientific). Each membrane section was blocked with 5% BSA (#GAUBSA01-64; Eurobio) in Tris Buffered Saline with Tween^®^-20 (TBS-T) 1X (#28360; Thermo Fisher Scientific) and incubated overnight at 4°C with gentle agitation with the following primary antibodies: anti-USB1 1/1,000 (#240421, RRID:AB_2909426; Abcam), anti-HA tag 1/2000 (#H6908, RRID:AB_260070; Sigma-Aldrich), anti-human SP1 (D4C3) 1/1,000 (#9389S, RRID:AB_11220235; Ozyme), anti-human HSP90 1/1,000 (#4874S, RRID:AB_2121214; Ozyme), and anti–β-actin 1/1,000 (sc-47778, #A2023, RRID:AB_626632; Santa Cruz Biotechnologies). After washing, membranes were incubated for 1 h with the appropriate HRP-conjugated secondary antibody (anti–mouse-HRP, #31430, RRID:AB_228307; Thermo Fisher Scientific or anti-rabbit-HRP, #31460, RRID:AB_228341; Thermo Fisher Scientific). Chemiluminescence detection was performed using the SuperSignal West Femto Maximum Sensitivity Substrate (Thermo Fisher Scientific) on a ChemiDoc XRS system (Bio-Rad). The resulting images were analyzed using Image Lab 4.0 software (Bio-Rad).

#### Immunofluorescence

Glass coverslips (ø 12 mm) have been sterilized and coated for 1 h at room temperature with 0.1 mg/ml Poly D lysine (#A3890401; Gibco). After rinsing the culture surface for three times with distilled water and letting it dry, 60,000 cells/condition of HEK293T cells expressing USB1-HA variants have been plated overnight. The following day, cells were washed once with PBS and fixed with 4% paraformaldehyde (PFA, #P6148; Sigma-Aldrich) for 15 min. After fixation, cells were rinsed with PBS and permeabilized using 0.5% Triton X-100 (Triton X-100, #086K0164; Sigma-Aldrich) in PBS for 10 min. To block nonspecific binding, the cells were incubated for 40 min in a blocking solution consisting of previously filtered 5% BSA (BSA Fraction V, #GAUBSA01-62; Sigma-Aldrich) in PBS. Primary antibodies were diluted in PBS with 5% BSA and incubated overnight at 4°C: anti-HA Rabbit 1/50 (#H6908, RRID:AB_260070; Sigma-Aldrich) and anti-SC35 1/200 (#Ab11826, RRID:AB_298608; Abcam). Following two washes with PBS, secondary antibodies were added, tailored to the isotype or species of the primary antibody: Goat anti-Mouse IgG Secondary Antibody, Alexa Fluor 555 (1/1,000, #A21422, RRID:AB_2535844; Thermo Fisher Scientific), Donkey anti-Rabbit IgG, Alexa Fluor 488 (1/1,000, #A21206, RRID:AB_2535792; Thermo Fisher Scientific) were incubated for 30 min at room temperature. After three final PBS washes with gentle agitation, the coverslips were mounted using ProLong Gold Antifade Mountant with DNA Stain DAPI (#P36935; Invitrogen). Z stacks were acquired on a Leica SP8 confocal microscope with a 63× NA1.4 oil objective (Plateforme d’Imagerie Cellulaire, SFR Necker) with the LAS X version 3.5.7.23225 (Leica Application Suite X) acquisition software. Imaging was performed on fixed cells, and acquisitions were conducted at room temperature. Images have been analyzed with Fiji (version 2.14.0/1.54f, RRID:SCR_002285).

For cell compartment intensity and morphology analysis, stacks were first projected in maximum intensity. A FIJI (26) (v 2.14.0, RRID:SCR_002285) macro and the BIOP (https://github.com/BIOP/ijl-utilities-wrappers) plugin of cellpose2 (27, 28) cyto2 model were used for nuclei and cytoplasm segmentation with the possibility of having manual corrections. Mean intensities in nuclei and cytoplasm for each cell have been measured. For this analysis, a total of 143 for USB1^WT^-HA and 149 for USB1^P44L^-HA cells were analyzed across three independent experiments. The results are presented to illustrate individual data points in a different color for each independent experiment. To allow the application of the normal law in statistics, a minimum of 25 cells were analyzed for each condition per replicate.

To assess signal overlap between SC-35 and USB1-HA variants in nuclei, we firstly denoised the stacks using the PureDenoise (29) plugin in a FIJI (RRID:SCR_002285) (26) macro. Next, we segmented the nuclei, SC-35 aggregates, and USB1-HA protein in 3D, employing a supervised voxel classification shallow learning method in Ilastik (30) (v1.4.0.post1). To identify only USB1-HA high-intensity signal, four different labels were used to train the Ilastik machine learning model: high, medium, low, and background. These annotations allowed the identification of what we defined as USB1-HA aggregates, which was then investigated for co-localization with the SC-35 signal. Pixel classification for the SC-35 signal was based on two labels: signal and background. Semiautomated quantification was executed in FIJI (RRID:SCR_002285) (26), and a threshold of 27 voxels was then applied to define an aggregate. A total of 56 for USB1^WT-^HA and 76 for USB1^P44L^-HA cells were analyzed across three independent experiments.

#### Northern blot and 3′ RACE analysis

To generate a ladder (67 bp) for the northern blot analysis, the U6 PCR product amplified starting from cDNA of control BEBVs (hU6_R 5′-GGA​ACT​CGA​GTT​TGC​GTG​TCA​TCC​TTG​CGC-3′ and T7_U6_F1 5′-TAA​TAC​GAC​TCA​CTA​TAG​GAA​TCT​AGA​ACA​TAT​ACT​AAA​ATT​GGA​AC-3′ primers) was purified from the 2% agarose gel following the manufacturer’s instructions (HighPure PCR Product Purification Kit, #11732676001; Roche). For *in vitro* transcription, Standard RNA Synthesis (#E2050; New England Biolabs) was used.

For RNA isolation, 10 million BEBVs were collected and washed once with PBS. RNeasy Midi Kit (#75144; Qiagen) was used according to the manufacturer’s instructions, including the DNAse I treatment. To remove 3′ terminal phosphate groups (if present), RNA was treated with T4 polynucleotide kinase (PNK) (31). Briefly, 15 μg of total RNA was incubated with 6 U T4 PNK (#M0201; New England Biolabs) in 100 mM Tris-HCl, pH 6.5, 100 mM magnesium acetate, and 5 mM β-mercaptoethanol in a final volume of 100 μl and incubated overnight at 37°C. RNA treated with PNK buffer only was included as a control. The enzyme was heat inactivated by incubating at 65°C for 20 min. Clean-up of RNA was performed with Monarch RNA Cleanup kit (#T2050L; New England Biolabs). RNA was then treated with *Escherichia coli* Poly(A) Polymerase (#M0276; New England Biolabs) and incubated at 37°C for 30 min. The enzyme was heat inactivated by incubating at 65°C for 20 min. Clean-up of RNA was performed with Monarch RNA Cleanup kit (#T2050L; New England Biolabs). 4–15 μg of RNA was heated at 95°C for 3 min and resolved in 10% Tris-borate-EDTA (TBE) Urea Gel (#EC68752BOX; Thermo Fisher Scientific) in TBE running buffer (#LC6675; Thermo Fisher Scientific) at 20 V the first hour and then increased by 5–10 V to reach 40 V overnight at 4°C. The buffer was changed in the middle of the run. RNA was transferred on Hybond-N^+^ membrane (#RPN303B; Cytiva) using the Trans-Blot Turbo transfer system (Bio-Rad) for 1 h at 25 V–1.0A. The membrane was briefly dried on a paper towel and cross-linked twice (face up) using a 254 nm UV cross-linker at 125 mJ/cm^2^. In the hybridization oven, the membrane was pre-hybridized in a tube with 10 ml ExpressHyb hybridization solution (#636833; Takara) for 40 min at 40°C. The pre-hybridization solution was removed, and the membrane was hybridized overnight at 40°C with 100 pmoles IR U6 probe (h_U6, 5′-GGA​ACT​CGA​GTT​TGC​GTG​TCA​TCC​TTG​CGC-3′ with 5′ IRDye 800CW). The probe was at 95°C for 5 min and then diluted in 10 ml ExpressHyb hybridization solution. The membrane was washed twice in a glass tray with 2× saline-sodium citrate (SSC, #T9172; Takara)-0.1% SDS (#0503; Sigma-Aldrich) at 110 rpm for 10 min at 40°C, dried with paper towels, and imaged on Li-Cor Odyssey CLX Scanner to detect emission. The probes were removed from the membrane by placing it in a glass tray and shaken with microwave-boiled 0.1x SSC-1% SDS-40 nM Tris-HCl, pH 8.0, at 110 rpm for 10 min at room temperature. The strip procedure was performed a second time prior to pre-hybridization and overnight 40°C hybridization with 100 pmoles IR 5S preheated probe (h_5S 5′-AAG​TAC​TAA​CCA​GGC​CCG​AC-3′, with 5′ IRDye 800CW).

For 3′ RACE experiments, 1 µg of PNK-PAP–treated RNA was used, according to the manufacturer’s instructions (FirstChoice RLM-RACE kit, #AM1700; Ambion). For reverse transcription, a 3′ RACE adapter with 2 additional As at the 3′ was used (5′-GCG​AGC​ACA​GAA​TTA​ATA​CGA​CTC​ACT​ATA​GGT12TAA-3′). 3′ RLM-RACE PCR was performed using U6 3′ RACE primer (5′-GGA​ATC​TAG​AAC​ATA​TAC​TAA​AAT​TGG​AAC-3′) and GoTaq DNA Polymerase (# M7845; Promega) with an annealing temperature of 55°C for 30 s. RACE products were purified with the High Pure PCR Product Purification Kit (#11732676001; Roche), cloned using the PCR Cloning Kit (#231122; QIAGEN) at 16°C for 2 h, and transformed into bacteria (One Shot MAX Efficiency DH5αTM-T1R Competent Cells, #12297-016; Thermo Fisher Scientific). UltraPure X-Gal (#15520-034; Invitrogen) was added on the top of the agar plates to allow blue/white screening to select transformants. At least 24 independent clones per sample in each experiment were picked. Wizard Plus SV Minipreps DNA Purification System (#A1460; Promega) was used to extract the DNA from each clone. For the following Sequencing BigDye reaction, M13_F 5′-GTA​AAA​CGA​CGG​CCA​GT-3′ was used as primer.

#### Quantitative PCR (qPCR) snRNA

Total RNA was isolated using Trizol with Phasemaker tubes in accordance with the manufacturer’s instructions (#A33250; Invitrogen). 6 μg of total RNA was treated with Recombinant DNase I (#2270A; Takara). Polyadenylation, reverse transcription, and qPCR were performed using the Mir-XTM miRNA qRT-PCR TB Green Kit (#638314; Takara) and U6 (#638314; Takara) and 5S RNAs primers (5S qPCR F 5′-GCC​ATA​CCA​CCC​TGA​ACG-3′ and 5S qPCR R 5′-GGT​ATT​CCC​AGG​CGG​TCT-3′). QuantStudio 3 Real-Time PCR System was used, and results were analyzed using the ΔΔCt method and normalized to 5S levels.

#### Co-immunoprecipitation

15 × 10^6^ HEK293T cells expressing USB1-HA (or EV as control) were washed once with PBS. After a centrifugation at 1,000 *g* for 5 min, 900 μl of ice-cold IP Lysis/Wash Buffer (Pierce Magnetic HA-Tag IP/Co-IP Kit, #88838; Thermo Fisher Scientific) completed with Protease/Phosphatase Inhibitor Cocktail (100X) (#5872S; Ozyme) was added and incubated on ice for 5 min with periodic mixing. Cells debris were removed by centrifugation (13,000 *g* for 10 min, at 4°C), and protein was quantified (Bradford assay). 25 μl of Pierce Anti-HA Magnetic Beads were washed with IP Lysis/Wash Buffer using a magnetic stand. 600 μg of protein was incubated with the pre-washed beads and shaken on a rotating platform overnight at 4°C. After removing the unbound sample, 300 μl of IP Lysis/Wash Buffer was added to the tube and gently mixed. At the end of co-immunoprecipitation wash step, additional washes were carried out to eliminate the detergent (three washes in PBS and one with ultrapure water; tubes were changed after each wash step). The immunoprecipitates were eluted by adding 100 μl of Laemmli Buffer 1X (#1610747; Bio-Rad) to the beads and incubated in a heat block at 95°C for 5 min 10% of the eluate was resolved in a western blot to check the quality of the sample, following the protocol above (Anti-human PLRG1 1/2500 [#A301-940A-1, RRID:AB_1548014; Thermo Fisher Scientific] and anti-HA tag 1/2,000 [#H6908, RRID:AB_260070; Sigma-Aldrich] were used as primary antibodies).

The remaining 90% of eluates were solubilized in 2× lysis buffer (4% SDS and 400 mM triethylammonium bicarbonate, pH 8.5) and boiled for 5 min at 95°C. Samples were also reduced and alkylated (10 mM TCEP and 50 mM chloroacetamide). The whole samples were digested using trypsin (Promega), and S-Trap Micro Spin Column was used according to the manufacturer’s protocol (ProtiFi). Peptides were then speed-vacuum dried. Nano-scale liquid chromatographic tandem mass spectrometry (nLC-MS/MS) analyses were performed on a Dionex U3000 RSLC nano-LC system coupled to a TIMS-TOF Pro mass spectrometer (Bruker Daltonik GmbH). After drying, peptides were solubilized in 10 μl of 0.1% TFA containing 10% acetonitrile (ACN). 1 μL was loaded from samples, concentrated, and washed for 3 min on a C18 reverse phase column (5 μm particle size, 100 Å pore size, 300 μm inner diameter, and 0.5 cm length, from Thermo Fisher Scientific). Peptides were separated on an Aurora C18 reverse phase resin (1.6 μm particle size, 100 Å pore size, 75 μm inner diameter, and 25 cm length mounted to the Captive nanoSpray Ionisation module, from IonOpticks) with a 1 h run time with a gradient ranging from 98% of solvent A containing 0.1% formic acid in MilliQ-grade H_2_O to 40% of solvent B containing 80% ACN and 0.085% formic acid in mQH2O. The mass spectrometer acquired data throughout the elution process and operated in DIA Parallel Accumulation and Serial Fragmentation (PASEF) mode with a 1.38 s/cycle, with Timed Ion Mobility Spectrometry (TIMS) enabled and a data-independent scheme with full MS scans in PASEF. Ion accumulation and ramp time in the dual TIMS analyzer were set to 100 ms each, and the ion mobility range was set from 1/K0 = 0.63 V s cm^−2^ to 1.43 V s cm^−2^. Precursor ions for MS/MS analysis were isolated in positive polarity with PASEF in the 400–1,200 m/z range by synchronizing quadrupole switching events with the precursor elution profile from the TIMS device. The mass spectrometry data were analyzed using DIA-NN version 1.8.1 (RRID:SCR_022865). The database used for in silico generation of spectral library was a concatenation of human sequences from the Swiss-Prot (release 2024-06) and a list of contaminant sequences. Oxidation of methionine was set as variable modification, carbamidomethylation of cysteine was set as permanent modification, and one trypsin misscleavage was allowed. Precursor false discovery rate (FDR) was kept below 1%. The “match between runs” and normalization options was not allowed. Quantification analysis was done using home R script. Log_2_ of protein intensities were calculated, then paired student tests were done on proteins showing 70% of paired valid values.

#### Injection of zebrafish embryos and phenotypic analysis

Zebrafish were raised and maintained according to established techniques (32) and to the European recommendations (33) and Italian regulations. All experimental procedures were performed according to Institutional Animal Care and Use Committee guidelines. Zebrafish *Tg(mpx:GFP)* (ZDB-TGCONSTRCT-070118-1) was kindly provided by Dr. Monica Beltrame (Università degli Studi di Milano Statale, Milano, Italy). Embryos were cultured in fish water containing 0.01% methylene blue to prevent fungal growth and staged according to morphological criteria. Embryonic ages were expressed as hours postfertilization and dpf.

Antisense morpholinos (MOs; Gene Tools, RRID:SCR_005663) designed against the acceptor splice site of the *usb1* IVS2 (SMO-A, 5′-GGA​TCA​TCT​GAA​ATT​TAG​GCA​GGA​A-3′) was used (20). Std-MO, which does not have a target in zebrafish embryos, was included to check for nonspecific effects due to the injection procedure (20). 10 µg of each pCS2^+^ plasmids (USB1 variants and mRFP1) were digested with 2 μl of NotI-HF enzyme (#R3189S; NEB) for 2 h at 37°C in a final volume of 100 μl. Digested and undigested plasmids were resolved in a 1% agarose gel to check the correct digestion of the plasmids. 1 µg of each digested and purified plasmid (GeneJET PCR purification Kit, #K0701; Thermo Fisher Scientific) was transcribed with the mMESSAGE mMACHINE (#AM1340; Thermo Fisher Scientific). The reaction was incubated for 3 h at 37°C, and the template DNA was digested by adding 1 μl of TURBO DNAse to the reaction (37°C for 15 min). mRNA was precipitated using lithium chloride overnight at −20°C and washed with ethanol, following the manufacturer’s instructions. RNA’s quality was checked by running a 1% agarose gel using a RiboRuler High Range RNA ladder (#SM1823; Thermo Fisher Scientific). RNA was heated at 70°C for 10 min before loading the gel. RNA was then quantified, aliquoted, and stored at −80°C until needed.

Needles were prepared with the P87 Flaming Brown Micropipette Puller. Morpholinos and mRNA were thawed, diluted in water, heated at 65°C for 10 min, and pressure-injected into 1–2-cell stage embryos using Eppendorf FemtoJet Micromanipulator 5171. For co-injections, SMO-A-RNA mix was prepared to deliver the two molecules within one single injection. Embryos were raised in fish water containing 0.01% methylene blue, dechorionated, and anesthetized with 0.016% tricaine (ethyl 3-aminobenzoate methanesulfonate salt; Sigma-Aldrich) before observations and picture acquisitions. Images of 2 dpf embryos were taken on a Leica MZ FLIII epifluorescence stereomicroscope equipped with a DFC 480 digital camera and LAS Leica imaging software (Leica). For each embryo, two pictures were taken: one at low magnification (5×) and one, zooming on the tail, at higher magnification (10×). Each picture was taken both in bright-field and using lasers at 488 or 590 nm to induce the excitation of GFP and mRFP1, respectively. Neutrophil count, pigmentation, and area of the tail were evaluated at 2 dpf and quantified with Fiji 2.3.0/1.52q (RRID:SCR_002285) software. Quantifications were performed in the same region of interest for each fish. This area goes from the anus to the end of the tail, where the caudal hematopoietic tissue is located (34).

Alcian blue staining was performed at 5 dpf as previously described (20). Larvae were anesthetized with tricaine, and up to 100 larvae were collected in a single 1.5-ml tube. 1 ml of 4% PFA (#P6148; Sigma-Aldrich) in PBS was added to fix tissues. Embryos were rocked at room temperature for 2 h, washed two times with PBS 1X, and dehydrated for 10 min with 1 ml EtOH 50% (diluted in distilled water) at room temperature. 1 ml of stain solution was added (part A: Alcian blue 8GX 0.02% [#A3157; Sigma-Aldrich], MgCl_2_ 40 mM [#M2670-100gr; Sigma-Aldrich], and ethanol 70%; part B: 0.5% alizarin red powder [#A5533; Sigma-Aldrich] in H_2_O) and incubated overnight at room temperature with rocking. The day after, embryos were rinsed with H_2_O and treated for 1 h with 20% glycerol and 0.25% KOH to remove pigmentation. Embryos were then incubated overnight in 50% glycerol and 0.25% KOH and stored in a 50% glycerol, 0.1% KOH at 4°C. Images of stained embryos were taken on a Leica MZ FLIII epifluorescence stereomicroscope equipped with a DFC 480 digital camera and LAS Leica imaging software (Leica). To facilitate the correct orientation of the embryos, they were positioned in an agar gel.

#### CD34^+^ isolation and differentiation

Human umbilical cord bloods were obtained from the Biological Resources Center of Saint Louis Hospital (Paris, France) in accordance with the ethical approval procedures (convention 2014/09/23). Mononuclear cells were isolated by density separation using SepMate PBMC Isolation Tubes (#85450; STEMCELL Technologies) and Lymphocyte Separation Medium (CMSMSL01; Eurobio). CD34^+^ cells were isolated using an indirect CD34 microbead kit and a separator (VarioMACS; Miltenyi Biotec), according to the manufacturer’s instructions. Cell purity was checked with a NovoCyte Flow Cytometer (Agilent). Only cells with a purity ≳95% were used for the following experiments. When indicated, 2 days after transduction, live GFP^+^ cells were sorted using FACS Buffer and 7-AAD as viability dye (BD FACSAria II Cell Sorter, 100 µm nozzle).

For CD34^+^*in vitro* liquid culture, cells were either maintained in pre-activation media (X-Vivo 15 medium [#BEBP02-061Q; Lonza] supplemented with 30% FBS [HyClone], 50 µg/ml of gentamycin, 300 ng/ml of SCF [300-01-100UG; Peprotech], 300 ng/ml of Fms-related tyrosine kinase 3 ligand [300-19-100UG; Peprotech], and 100 ng/ml of thrombopoietin [300-18-100UG; Peprotech]) or cultured in X-Vivo 15 medium supplemented with 30% FBS, 50 µg/ml of gentamycin, 100 ng/ml of SCF, and 100 ng/ml of G-SCF (300-23-10UG; Peprotech). After 8 or 14 days of differentiation, cultures were stained for 30 min using a combination of anti–CD34-APC (130-113-176, RRID:AB_2726003; Miltenyi), anti–CD11b-BV785 (301346, RRID:AB_2563794; BioLegend), anti–CD14-PECy7 (562698, RRID:AB_2737729; BD Biosciences), and anti–CD15-PE (IM1954U, RRID:AB_10638572; Beckman Coulter). Before the acquisition, 7-AAD staining was added, and cells were analyzed by NovoCyte Flow Cytometer (Agilent). When indicated, Epredia Cytocentrifuge Cytospin 4, Cytospin 4 (Thermo Fisher Scientific) was used to prepare 20,000 cells/condition for the MGG staining: it was performed via an automatic stainer (Hôpital Necker Enfants Malades, DMU BioPhyGen, Laboratoire d'Onco-Hematologie), and images were taken with an inverted microscope (DM1RB; Leica) at 10×.

CD34^+^ cell differentiation was also evaluated by clonal assay in methylcellulose (MethoCult H4435 or H4535), as previously described (35). Around 1,000 cells were gently mixed with 1 ml of MethoCult methylcellulose colony assay medium and were cultured for up to 14 days in 6-well plates at 37°C in humidified 5% CO_2_. Colonies were counted on day 10 (for #H4535) and day 14 (for #H4435) and classified according to the morphology and color of the colony using an inverted microscope (DM1RB; Leica) at 10×.

#### Protein sequence alignment and USB1 protein structure

For sequence alignment of human USB1 homologs from various species, protein sequences were extracted from the Ensembl Genome Browser (RRID:SCR_002344) and aligned using Clustal Omega (RRID:SCR_001591) and Jalview (RRID:SCR_006459). Shading intensity indicates the degree of amino acid identity. Accession numbers and transcript/protein identifiers from various biological databases were collected for orthologs across species. NCBI Reference Sequence: *Drosophila* (NP_649911.1), zebrafish (NP_001003460.1), *Xenopus* (NP_001079479.1), rat (NP_001014035.1), mouse (NP_598715.2), chimpanzee (XP_003315166.1), human (NP_078874.2), *Nomascus* (XP_012360491.1), *Saccharomyces cerevisiae* (NP_013233.1). Ensembl transcript identifiers: *Tetraodon* (ENSTNIT00000000453.1), chicken (ENSGALT00010052013.1), dog (ENSCAFT00000013577.5). GenBank: *Candida glabrata* (CAG62512.1)..1).

#### Statistical analysis

Ordinary one-way ANOVA and unpaired *t* test were performed using GraphPad Prism version 10.2.3, https://www.graphpad.com (RRID:SCR_002798). Gene ontology enrichment analysis was performed with ShyniGO 0.741, filtering with P value cutoff (FDR) 0.05 (18).

#### Artificial intelligence

Microsoft 365 Copilot was occasionally used to polish, condense, and edit the writing of the manuscript.

#### Online supplemental material

Fig. S1 shows the structural analysis of the P44L mutation in the USB1 protein. Fig. S2 presents the USB1^WT^ and USB1^P44L^ interactome compared to the EV, gene ontology enrichment analysis, together with illustrative images presenting the pixel classification used in the Ilastik model. Fig. S3 includes *in vitro* liquid culture differentiation assays. In Fig. S4, the results of ectopic expression of USB1^P44L^ in zebrafish are reported. In Table S1, the co-immunoprecipitation data are listed.

## Supplementary Material

Table S1 lists the co-immunoprecipitation data.

SourceData F2 is the source file for Fig. 2.

SourceData F3 is the source file for Fig. 3.

SourceData F4 is the source file for Fig. 4.

## Data Availability

All data are available in the published article and its online supplemental material. Fig. 4 C was obtained by filtering openly available proteomics data associated with a previous independent publication by Yuxin Xie et al. (17) and downloaded from the PRIDE-Proteomics Identification Database under accession numbers PXD056172.
