# Supplementary figures and images for: A heterozygous USB1 variant linked to immunodeficiency
Source: J Hum Immun. 2025 Oct 22;1(4):e20250110. doi: 10.70962/jhi.20250110 (PMC12851572; doi:10.70962/jhi.20250110)

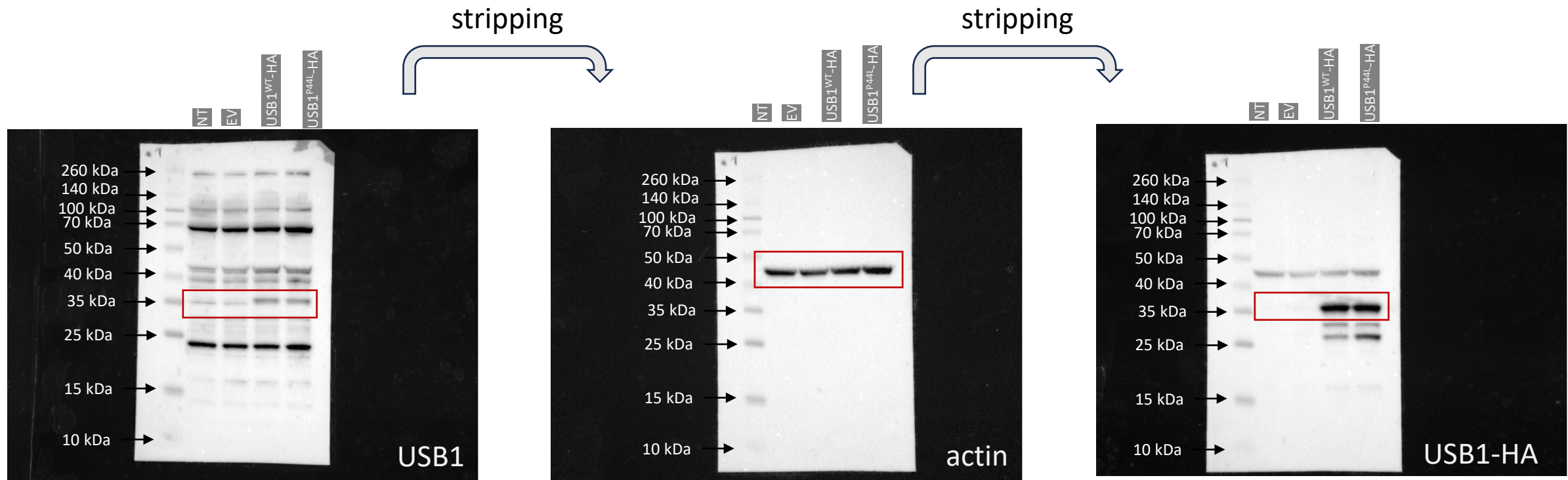

stripping

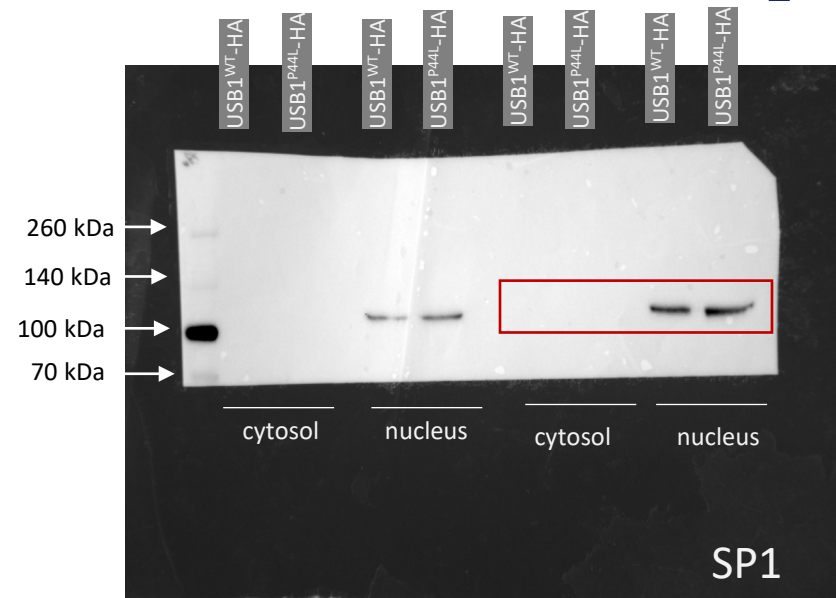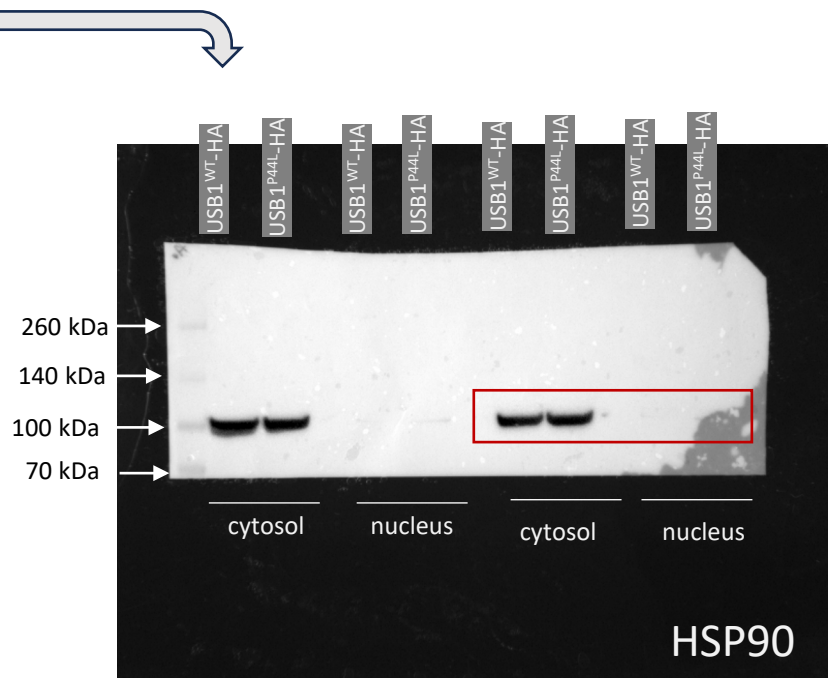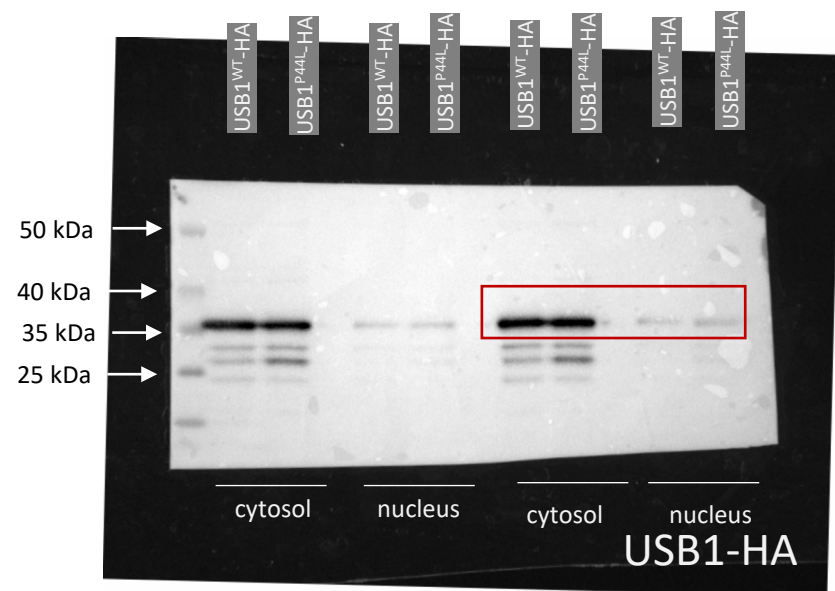

Supplement: SourceData F2 — is the source file for Fig. 2. [file jhi_20250110_sourcedataf2.pdf]

stripping

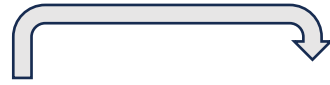

| ctrl |   |   | USB1-/- |   |   | P1 |   |   |          |
|------|---|---|---------|---|---|----|---|---|----------|
| -    | + | - | -       | + | - | -  | + | - | PNK      |
| -    | + | + | -       | + | + | -  | + | + | PNK buff |

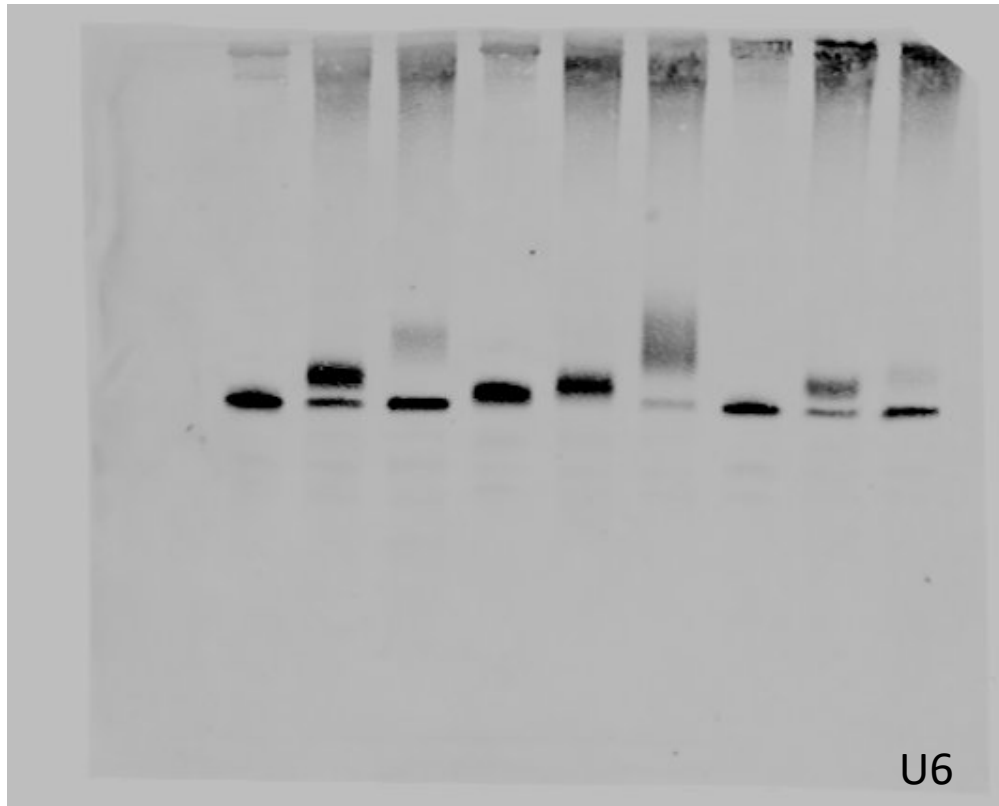

| ctrl |   |   | USB1-/- |   |   | P1 |   |   |          |
|------|---|---|---------|---|---|----|---|---|----------|
| -    | + | - | -       | + | - | -  | + | - | PNK      |
| -    | + | + | -       | + | + | -  | + | + | PNK buff |

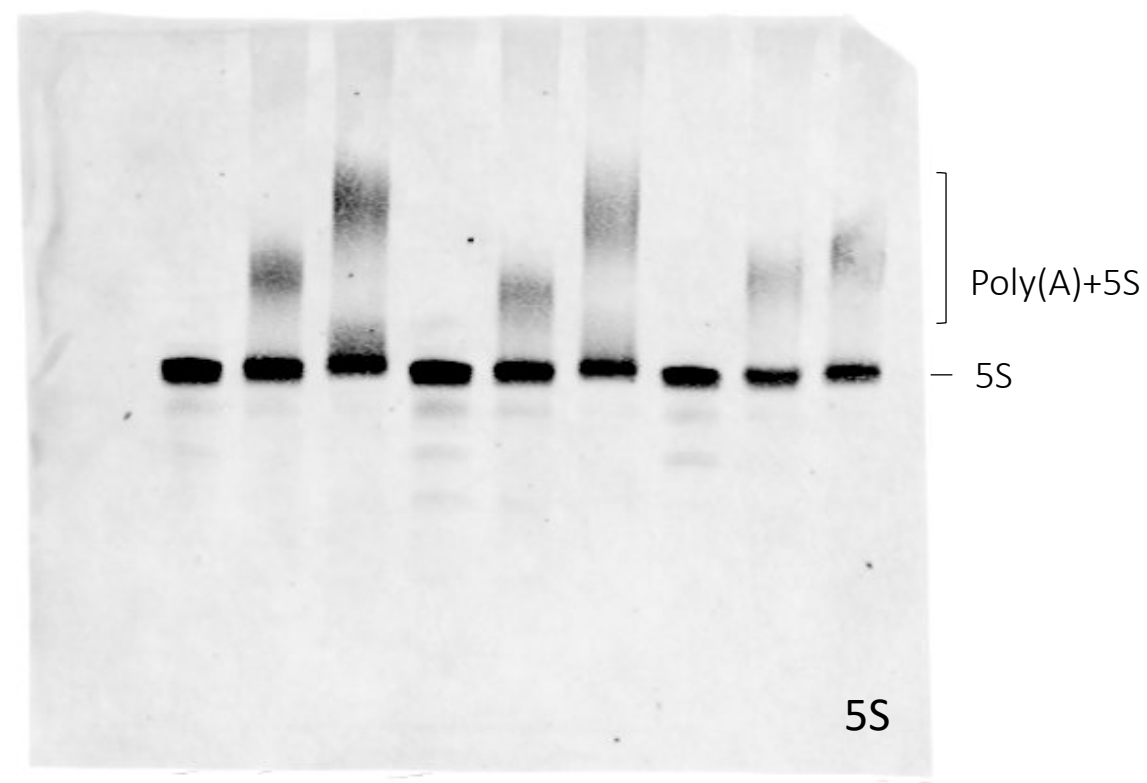

stripping

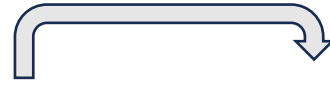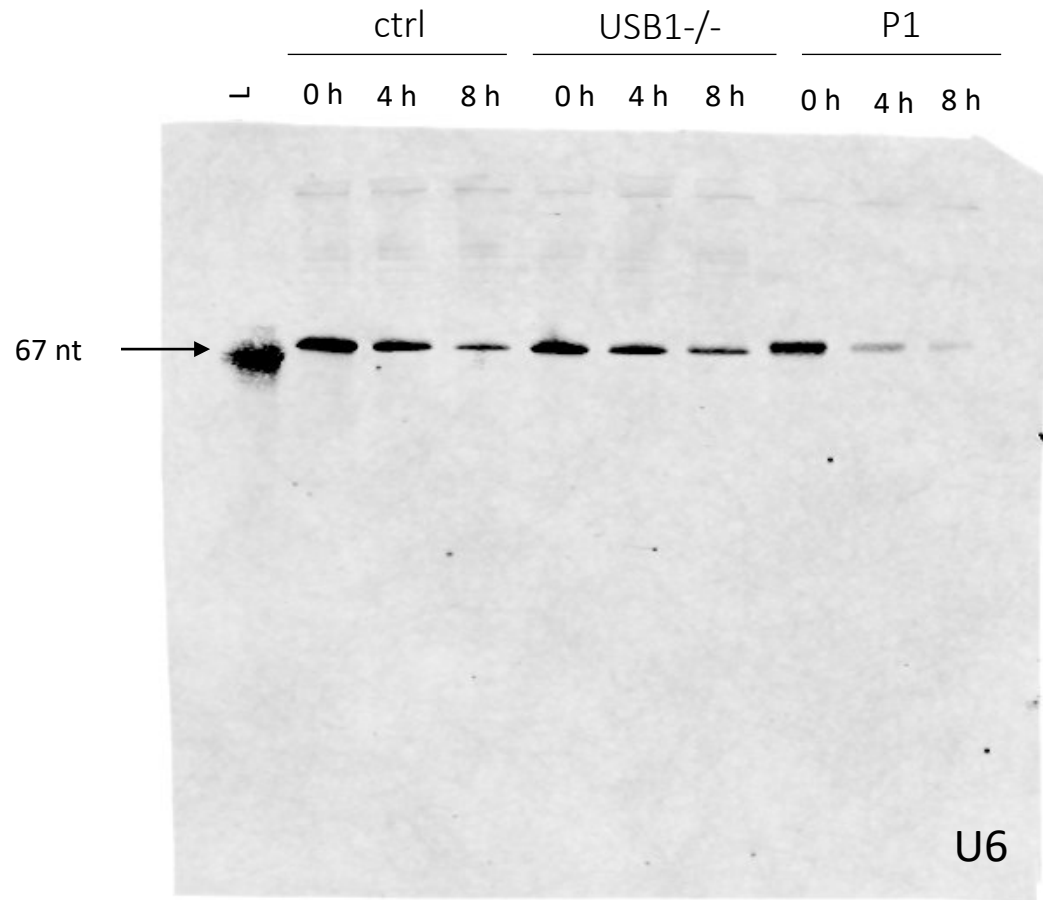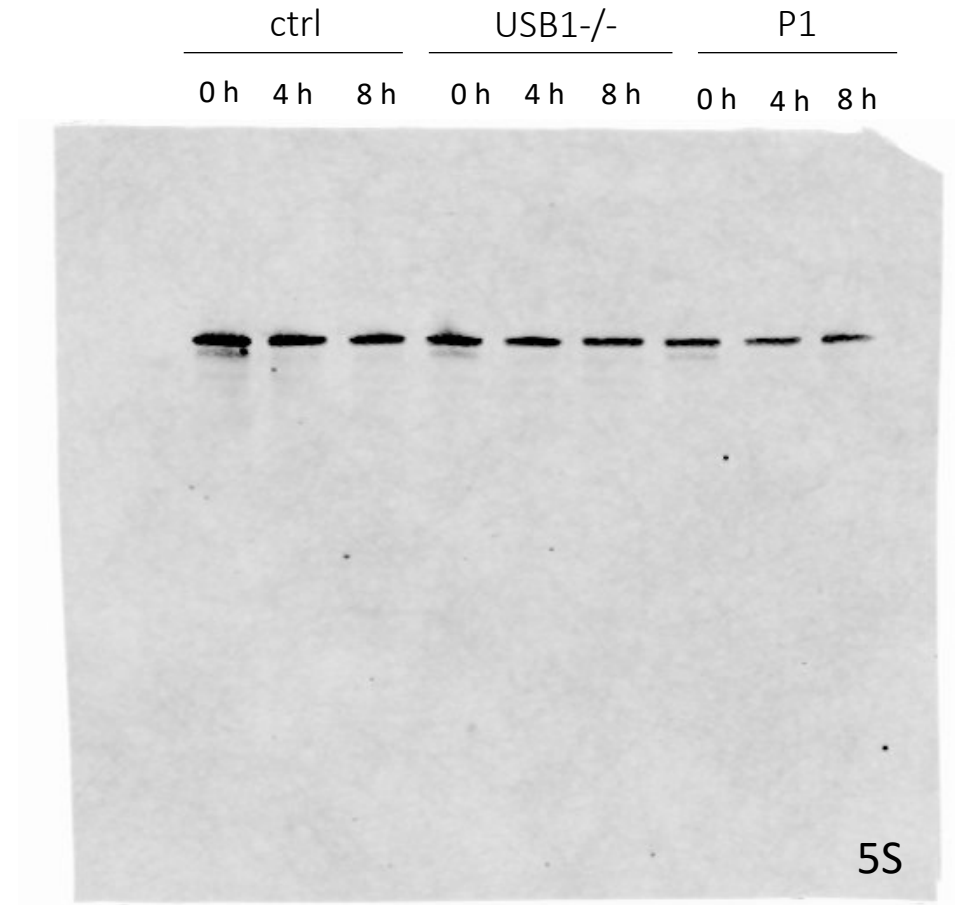

Supplement: SourceData F3 — is the source file for Fig. 3. [file jhi_20250110_sourcedataf3.pdf]

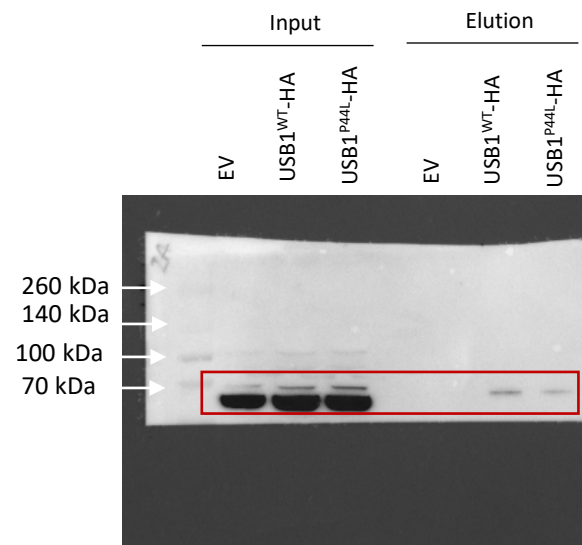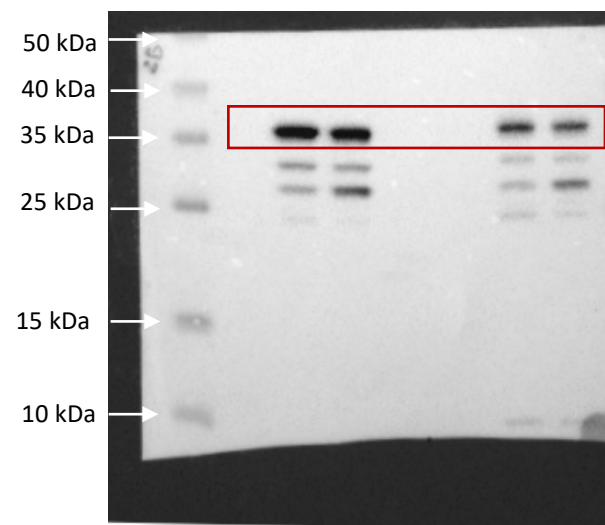

Supplement: SourceData F4 — is the source file for Fig. 4. [file jhi_20250110_sourcedataf4.pdf]
